# The RNA helicase DDX3X is an essential mediator of innate antimicrobial immunity

**DOI:** 10.1371/journal.ppat.1007397

**Published:** 2018-11-26

**Authors:** Daniel Szappanos, Roland Tschismarov, Thomas Perlot, Sandra Westermayer, Katrin Fischer, Ekaterini Platanitis, Fabian Kallinger, Maria Novatchkova, Caroline Lassnig, Mathias Müller, Veronika Sexl, Keiryn L. Bennett, Michelle Foong-Sobis, Josef M. Penninger, Thomas Decker

**Affiliations:** 1 Max F. Perutz Laboratories, Department of Microbiology, Immunobiology and Genetics, University of Vienna, Vienna Biocenter, Vienna, Austria; 2 Institute of Molecular Biotechnology of the Austrian Academy of Sciences (IMBA), Vienna Biocenter, Vienna, Austria; 3 Research Institute of Molecular Pathology (IMP), Vienna Biocenter (VBC), Vienna, Austria; 4 Institute of Animal Breeding and Genetics, University of Veterinary Medicine Vienna, Vienna, Austria; 5 Institute of Pharmacology and Toxicology, University of Veterinary Medicine Vienna, Vienna, Austria; 6 CeMM Research Center for Molecular Medicine of the Austrian Academy of Sciences, Vienna, Austria; University of Pennsylvania, UNITED STATES

## Abstract

DExD/H box RNA helicases, such as the RIG-I-like receptors (RLR), are important components of the innate immune system. Here we demonstrate a pivotal and sex-specific role for the heterosomal isoforms of the DEAD box RNA helicase DDX3 in the immune system. Mice lacking DDX3X during hematopoiesis showed an altered leukocyte composition in bone marrow and spleen and a striking inability to combat infection with *Listeria monocytogenes*. Alterations in innate immune responses resulted from decreased effector cell availability and function as well as a sex-dependent impairment of cytokine synthesis. Thus, our data provide further *in vivo* evidence for an essential contribution of a non-RLR DExD/H RNA helicase to innate immunity and suggest it may contribute to sex-related differences in resistance to microbes and resilience to inflammatory disease.

## Author summary

The establishment of innate immunity to pathogens requires cells to sense microbial molecules and to initiate a de novo transcription-based antimicrobial response. With the identification of Rig I and Mda5, two RNA helicases were shown to serve as pivotal receptors of viral RNA. Subsequently, a considerable number of RNA helicases were proposed to function as sensors or signal transducers for both microbial RNA and DNA. X-chromosome-encoded RNA helicase DDX3X was discovered as an interactor of the S/T kinase TBK1 which regulates the production of type I Interferons (IFN-I). However, the importance of DDX3X for innate immunity in an organismic context remained elusive. Here we describe and analyze mice lacking DDX3X in hematopoietic cells. We show contributions of DDX3X to hematopoiesis and a striking loss in resistance against *Listeria monocytogenes*. Our data reveal that DDX3X is critically involved in enhancing the expression of numerous antimicrobial genes. Consistently, production of important cytokines such as IL12 or IFNγ is reduced. Furthermore, DDX3X-deficient macrophages show reduced ability to restrict *L*. *monocytogenes* growth. Owing to partial redundancy with its close Y-chromosomal homologue, DDX3Y, the observed effects differ between mouse sexes. Thus, DDX3X may contribute to sex differences in immunity to pathogens and inflammatory disease.

## Introduction

Upon infection, germline-encoded pattern recognition receptors (PRRs) located on the surface of cells, in endosomal compartments and throughout the cytosol initiate an array of signaling cascades that culminate in the production of type I interferons (IFN-I), pro-inflammatory cytokines and chemokines. These cytokines establish an inflammatory response and an antimicrobial state restraining the spread of the infectious agent.

The discovery of Rig-I-like receptors (RLR) as sensors of viral RNA sparked considerable interest in the role of other DExD/H RNA helicases as innate modulators of antimicrobial immune responses [1,2]. DExD/H helicases not belonging with typical RLR also contribute to innate immunity in experimental animals, as recently demonstrated for DDX41 which acts in dendritic cells to limit retroviral growth [3]. We and others have identified the RNA helicase DDX3X as a regulator of IFN-I transcription in cells infected with viruses or with the intracellular bacterial pathogen *Listeria monocytogenes* [4,5]. DDX3X belongs to the DEAD-box RNA helicase superfamily 2 [6] that has widespread functions in RNA metabolism, including transcription, RNA processing, splicing, decay and translation [7,8]. Moreover, DDX3X is implicated in cellular processes such as apoptosis, cell cycle regulation and tumorigenesis [9]. Deletion of DDX3X in all embryonic tissues causes the death of male embryos at an early postimplantation stage. By contrast, male embryos with epiblast-restricted DDX3X deletion die around E11.5 with widespread occurrence of apoptotic cells and expression of DNA damage markers [10]. This is most likely a direct consequence of a disturbed cell cycle in embryonic tissue lacking DDX3X. This view is further supported by a study investigating the role of DDX3X in early mouse development using siRNA-mediated knockdown [11].

A homologue of DDX3X called DDX3Y is encoded by the non-recombining region of the Y-chromosome. DDX3X and DDX3Y share around 90% homology. While DDX3X is ubiquitously expressed, DDX3Y protein expression was originally thought to be confined to the male germline [12]. More recent proteomic databases list DDX3Y in cells of the immune system, including T-cells, B-cells and NK-cells. Their high degree of similarity supports the idea that DDX3X and DDX3Y are functionally redundant [13]. The heterosomal origin of the DDX3 isoforms suggests they may contribute to sex-related differences in immunity to microbes, the ability to resolve inflammation and the propensity to develop autoinflammatory syndromes [14–16].

Several studies point to an ambiguous role of DDX3X in viral infections. On the one hand, it may promote replication of viruses like HIV or HCV [17–22]. On the other hand, DDX3X stimulates the production of antiviral IFN-I [23,24]. Antimicrobial pathways leading to IFN-I synthesis converge at two related S/T kinases, TBK1 and IKKε. DDX3X interacts with TBK1 and serves as its substrate [4]. It also interacts with IKKε (Schröder *et al*, 2008; Gu *et al*, 2013), the other non-canonical IKK kinase responsible for phosphorylation and activation of the interferon regulatory factors (IRF) 3 and 7 that control *Ifnb* gene transcription. In addition to its function downstream of TBK1/IKKε, DDX3X reportedly associates with the adaptor protein MAVS which localizes upstream of TBK1 and IKKε and supports signal transduction by the two DExH helicases RIG-I and MDA-5. In this context DDX3X was shown to sense viral RNA and to supplement the function of RIG-I and MDA-5 in the early phase of infection [25]. Strengthening its importance for antiviral immunity, a recent report lists DDX3X among genes conferring intrinsic antiviral immunity to stem cells [26].

Like viruses, the Gram-positive bacterium *Listeria monocytogenes* (Lm) is a potent inducer of IFN-I [27]. After phagocytosis by macrophages, Lm escapes to the cytosol via the secretion of listeriolysin O (LLO) that disrupts the phagosomal membrane [28,29]. The innate response to Lm is characterized by intracellular effector mechanisms as well as the secretion of many pro-inflammatory chemokines and cytokines, among them the IFN-I. Cytoplasmic nucleic acids derived from Lm were shown to trigger strong induction of the IFN-I genes. Lm DNA promotes IFN-I expression through the DNA sensors cGAS and IFI16, the adaptor molecule STING, as well as TBK1 kinase and its downstream targets IRF3 and IRF7 [30,31]. In addition to DNA, Lm RNA was implicated in the induction of type I IFNs via the cytosolic receptor RIG-I [32]. Knock-down of DDX3X phenocopies the silencing of TBK1 on *Ifnb* gene induction after Lm infection [4], emphasizing the general importance of DDX3X for the TBK-IRF3 axis. Despite the fact that IFN-I are crucial for protective antiviral responses, their impact on Lm infection appears to be detrimental for the host as evidenced by the observation that mice lacking the receptor for type I IFNs (IFNAR) are more resistant to parenteral infection with Lm [33–35]. Unlike IFN-I, the type II interferon (IFNγ) is strongly associated with protective innate immunity against Lm [36,37]. Rapid production of IFNγ is essential and has been attributed to innate lymphocyte responses that include natural killer cells [38]. The resistance against Listeria provided by IFNγ is strongly associated with its role as a macrophage-activating cytokine [39].

Here we report a crucial role of DDX3X in the innate immune responses of cells and mice. We show that besides its role in the regulation of the TBK1-IRF3 axis, DDX3X controls the NFκB signaling pathway and has a profound impact on inflammatory cytokine production. DDX3Y, either alone or together with additional Y-chromosomal genes, partially compensates for the loss of the *Ddx3x* gene, as homozygous female cells and mice show more severe loss-of-function phenotypes. Mice lacking DDX3X in the hematopoietic system show alterations of bone marrow and splenic cell populations and are highly susceptible to Lm infection. Our data thus demonstrate a vital role of the sex-specific DDX3 isoforms in innate immunity to Listeria.

## Results

**Fig 1:**
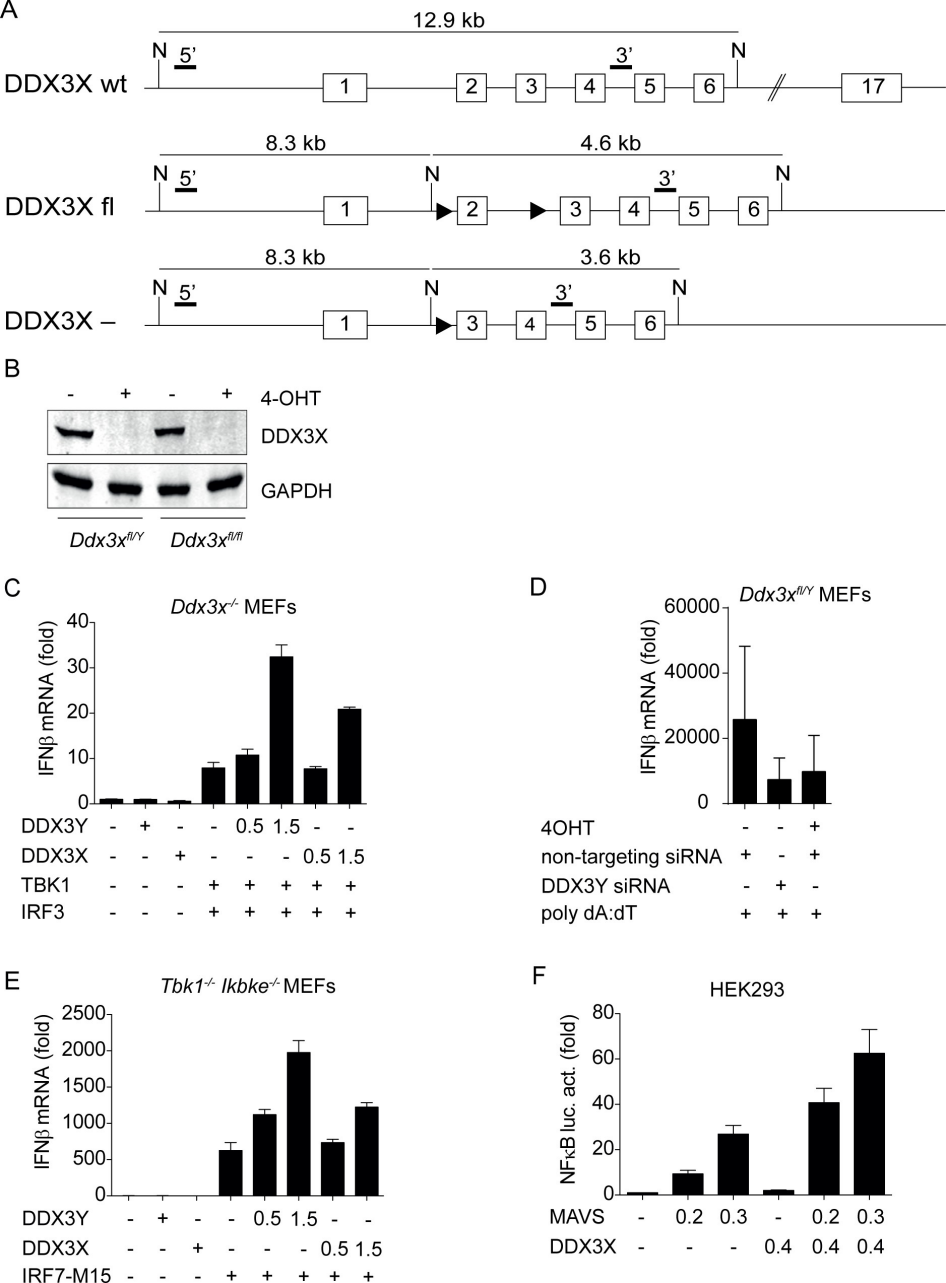
Generation of *Ddx3x*^*fl/fl*^ mice, analysis of DDX3X and DDX3Y activity in fibroblasts from gene-targeted mice. **A** Targeting strategy. Black triangles indicate loxP sites, black bars the position of probes used to confirm deletion by Southern blot. **B** Western blot showing the complete absence of DDX3X protein in bone marrow-derived macrophages from *Ddx3x*^fl/fl Cre*ERT2*^ female and *Ddx3x*^fl/y Cre*ERT2*^ male mice after treatment with 4-OHT during mCSF-mediated differentiation. **C** Mouse embryonic fibroblasts (MEFs) derived from female *Ddx3x*^fl/fl Cre*ERT2*^ mice were treated with 4-OHT to delete *Ddx3x*. The cells were transiently transfected with 1 μg IRF3, 1 μg TBK1 and increasing amounts of DDX3Y and DDX3X (numbers denote μg transfected plasmid). One day after transfection, RNA was isolated and *Ifnb* expression was determined by qPCR. Bars represent the mean value +/-SD of technical replicates. Due to variations in transfection efficiencies across different experiments one representative experiment of at least three biological replicates is shown. **D** Mouse embryonic fibroblasts (MEFs) derived from male Ddx3x^fl/y CreERT2^ mice were treated with 4-OHT to remove DDX3X, or with Ddx3y siRNA as indicated. Subsequently the cells were transfected with 10 μg of poly (dA:dT). 4h after transfection, RNA was isolated and Ifnb expression was determined by qPCR. Bars represent the mean value +/-SD of 3 biological replicates. **E** *Tbk1/Ikkε*-deficient MEFs were transfected with 1 μg constitutively active IRF7-M15 and with increasing amounts of DDX3Y and DDX3X as indicated (right). Expression levels were normalized to *Gapdh* and to the expression level of cells transfected with empty vector. Bars represent the mean value +/-SD of technical replicates. Due to variations in transfection efficiencies across different experiments one representative experiment of at least three biological replicates is shown. **F** HEK293 cells were transfected with an NFκB reporter gene and the NFκB pathway was stimulated by co-transfecting different amounts of the adapter protein MAVS. The effect of DDX3X was assessed by additional co-transfection of a DDX3X expression plasmid. Bars represent the mean value +/-SD of technical replicates. Due to variations in transfection efficiencies across different experiments one representative experiment of at least three biological replicates is shown. Statistical analysis was carried out using unpaired, two-tailed Student's *t*-test. Mean and standard error of the mean (SEM) are indicated in the graphs. *P<0.05, **P<0.01, ***P<0.005.

To investigate DDX3X activity in the immune system we introduced loxP sites flanking exon 2 of the DDX3X locus into the mouse genome (*Ddx3x*^*fl/fl*^; Fig 1A). Deletion of DDX3X in bone marrow-derived macrophages (BMDM) by means of tamoxifen (4-OHT) -inducible Cre recombinase resulted in complete loss of DDX3X protein (Fig 1B). Complete Cre-mediated deletion of DDX3X in mice failed to produce viable offspring, consistent with impaired blastocyst formation and early embryonic lethality reported by others [10,11]. Vav-iCre -mediated deletion of DDX3X in the hematopoietic system allowed the development of male mice (*Ddx3x*^*fl/y Vav-iCre*^). In contrast, homozygous female offspring (*Ddx3x*^*fl/fl Vav-iCre*^) was not obtained. This emphasizes an important role of DDX3 isoforms in hematopoiesis and suggests that the Y chromosome contains genes that compensate for DDX3X deficiency to the point of ensuring survival and an absence of overt phenotypic abnormalities of unchallenged animals. The Y-chromosomal DDX3X homologue DDX3Y is an obvious candidate for this rescue, either alone or in combination with other Y-chromosomal genes. Consistent with an overlapping spectrum of activities, DDX3Y enhanced *Ifnb* gene expression in an almost identical manner to DDX3X when introduced into *Ddx3x*-deficient MEFs by transfection (Fig 1C) and knockdown of DDX3Y decreased poly (dA:dT)-stimulated IFNβ expression, similar to the tamoxifen-mediated knockout of DDX3X in fibroblasts derived from *Ddx3x*^fl/y-CreERT2^ mice (Fig 1D). Interestingly, both DDX3X and DDX3Y enhanced the activity of a constitutively active IRF7 variant, IRF7-M15 [40], in fibroblasts lacking both TBK1 and IKKε kinases (Fig 1E). This suggests that TBK1/IKKε-mediated phosphorylation is dispensable for the ability of DDX3X/Y to enhance IFNβ synthesis, or that fibroblasts express some constitutive DDX3X/Y kinase activity. Of further note, DDX3X was able to enhance the activity of an NFκB reporter gene (Fig 1F), suggesting its impact on innate immune responses may extend beyond the IRF3/7 pathway.

### Consequences of DDX3X deficiency for innate immunity against pathogens

**Fig 2:**
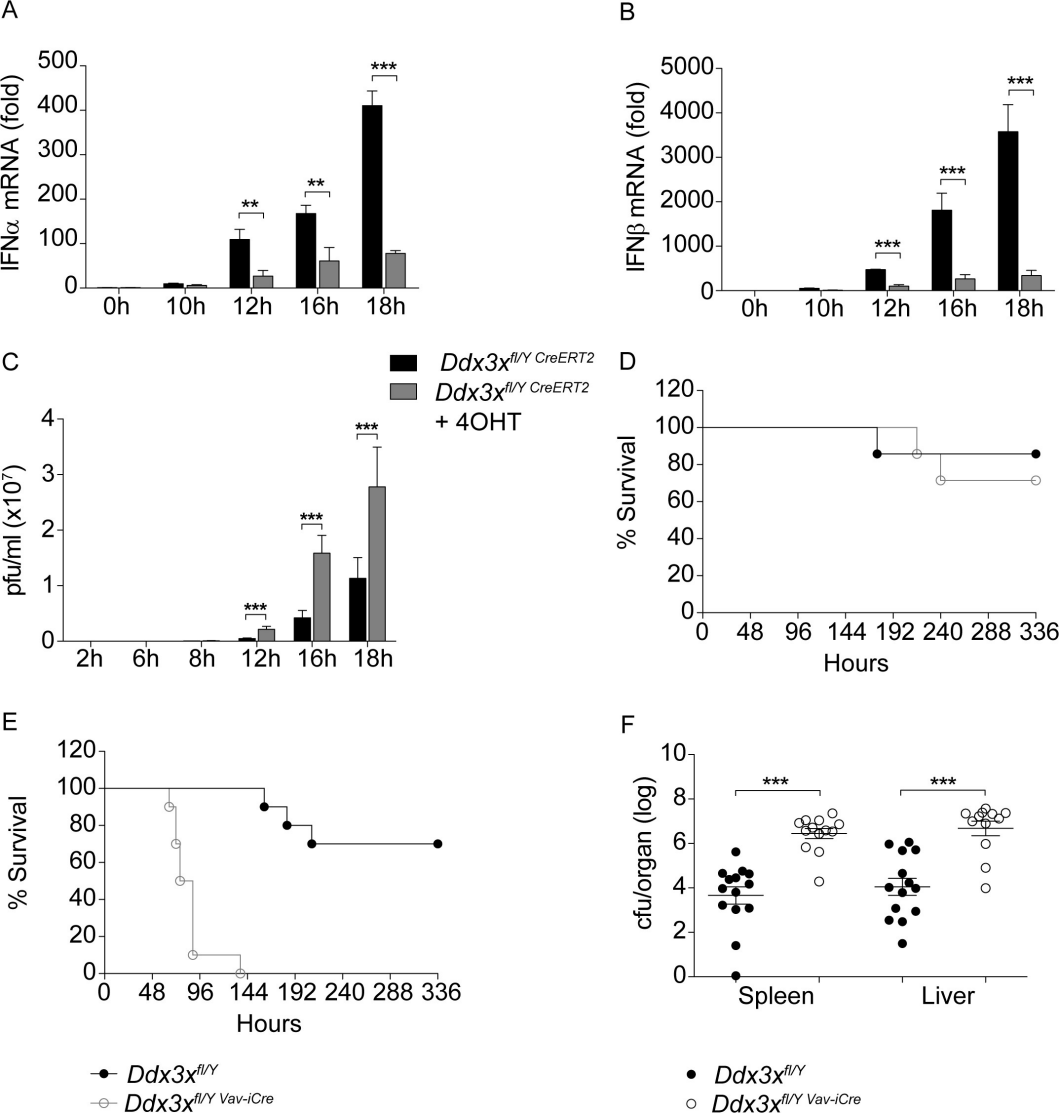
Innate immunity of DDX3X-deficient cells and of *Ddx3x*^fl/y *Vav-iCre*^ mice to VSV and *Listeria monocytogenes infection*. **A, B** WT MEFs, or MEFs derived from *Ddx3x*^fl/y CreERT2^ mice and treated with 4-OHT to delete *Ddx3x* were infected with VSV at an MOI of 0,1. After the indicated periods, RNA was isolated and pan-Ifna (A) or Ifnb (B) mRNA expression was determined by qPCR. Lines represent the mean +/- standard deviation (SD). Statistical significance was calculated using the unpaired, two-tailed Student's *t*-test. *P<0.05, **P<0.01, ***P<0.005. **C** Supernatants from cells infected with VSV at an MOI of 0.1 were collected and viral loads at the indicated times were measured by plaque-forming unit (pfu) assay. Lines represent the mean +/- standard deviation (SD). Statistical significance was calculated using the unpaired, two-tailed Student's *t*-test. *P<0.05, **P<0.01, ***P<0.005. **D** *Ddx3x*^fl/y^ and *Ddx3x*^fl/y *Vav-iCre*^ mice (n = 7) were infected intravenously with 10^6^ pfu of VSV. Survival was monitored for the indicated period of time. Statistical significance was calculated using the Log-rank (Mantel-Cox) test and Gehan-Breslow-Wilcoxon test. Differences between mouse genotypes did not reach statistical significance. **E** *Ddx3x*^fl/y^ and *Ddx3x*^fl/y *Vav-iCre*^ mice (n = 10) were injected intraperitoneally with 1,6x10^5^ CFU *L*. *monocytogenes* (strain EGD). Survival was monitored daily for the indicated period. Statistical significance was calculated using the Log-rank (Mantel-Cox) test (P-value: <0,0001) and Gehan-Breslow-Wilcoxon Test (P-value: <0,0001). **F** *Ddx3x*^fl/y^ mice (n = 14) and *Ddx3x*^fl/y *Vav-iCre*^ mice (n = 13) were injected intraperitoneally with 8x10^4^ CFU *L*. *monocytogenes* (strain EGD). After 72 h, mice were sacrificed and the bacterial load was determined in spleen and liver. Pooled data from three independent experiments is shown. Lines represent the mean +/- standard error of the mean (SEM). P-values were calculated using the unpaired, two-tailed Student's *t*-test. *P<0.05, **P<0.01, ***P<0.005.

Based on the role of DDX3X in IFN-I synthesis we investigated whether loss of the helicase causes a defect in innate immunity to virus. In male fibroblasts, derived from *Ddx3x*^fl/y-CreERT2^ mice and rendered DDX3X-deficient by treatment with tamoxifen, we observed reduced synthesis of IFNα as well as IFNβ mRNA following infection with vesicular stomatitis virus (VSV; Fig 2A and 2B). In accordance with this, VSV replicated to higher numbers in DDX3X-ablated cells compared to controls that had not been treated with tamoxifen (4OHT; Fig 2C). Surprisingly however, *Ddx3x*^*fl/y Vav-iCre*^ mice infected i.v. with VSV showed only a marginal reduction of viral clearance, in spite of the fact that hematopoietic cells contribute to IFN synthesis when i.v. injection is chosen as infection route (Fig 2D; [41,42]). This result suggests that any defect in IFN-I synthesis caused by deletion of DDX3X in hematopoietic cells can be compensated and that VSV infection does not reveal a major defect in the DDX3X-deficient innate immune system.

To further characterize the role of DDX3X in immune responses, we subjected *Ddx3x*^*fl/y Vav-iCre*^ mice to intraperitoneal (i.p.) infection with the intracellular bacterial pathogen *Listeria monocytogenes* (Lm). Compared to *Ddx3x*^*fl/y*^ control animals, *Ddx3x*^*fl/y Vav-iCre*^ mice were highly susceptible to Lm. All knockout animals succumbed to infection within 6 days, whereas most control animals survived the observation period (Fig 2E). In line with this, the bacterial burdens in spleens and livers of *Ddx3x*^*fl/y Vav-iCre*^ animals were strongly increased three days after i.p. infection (Fig 2F). IFN-I deficiency increases the innate resistance of mice against Lm [33–35]. Therefore, pro-survival effects of reduced IFN-I synthesis in *Ddx3x*^*fl/y Vav-iCre*^ animals are clearly overwhelmed by immune defects that weaken host resistance.

### Mice lacking DDX3X in hematopoietic cells have reduced numbers of lymphocytes and NK cells

**Fig 3:**
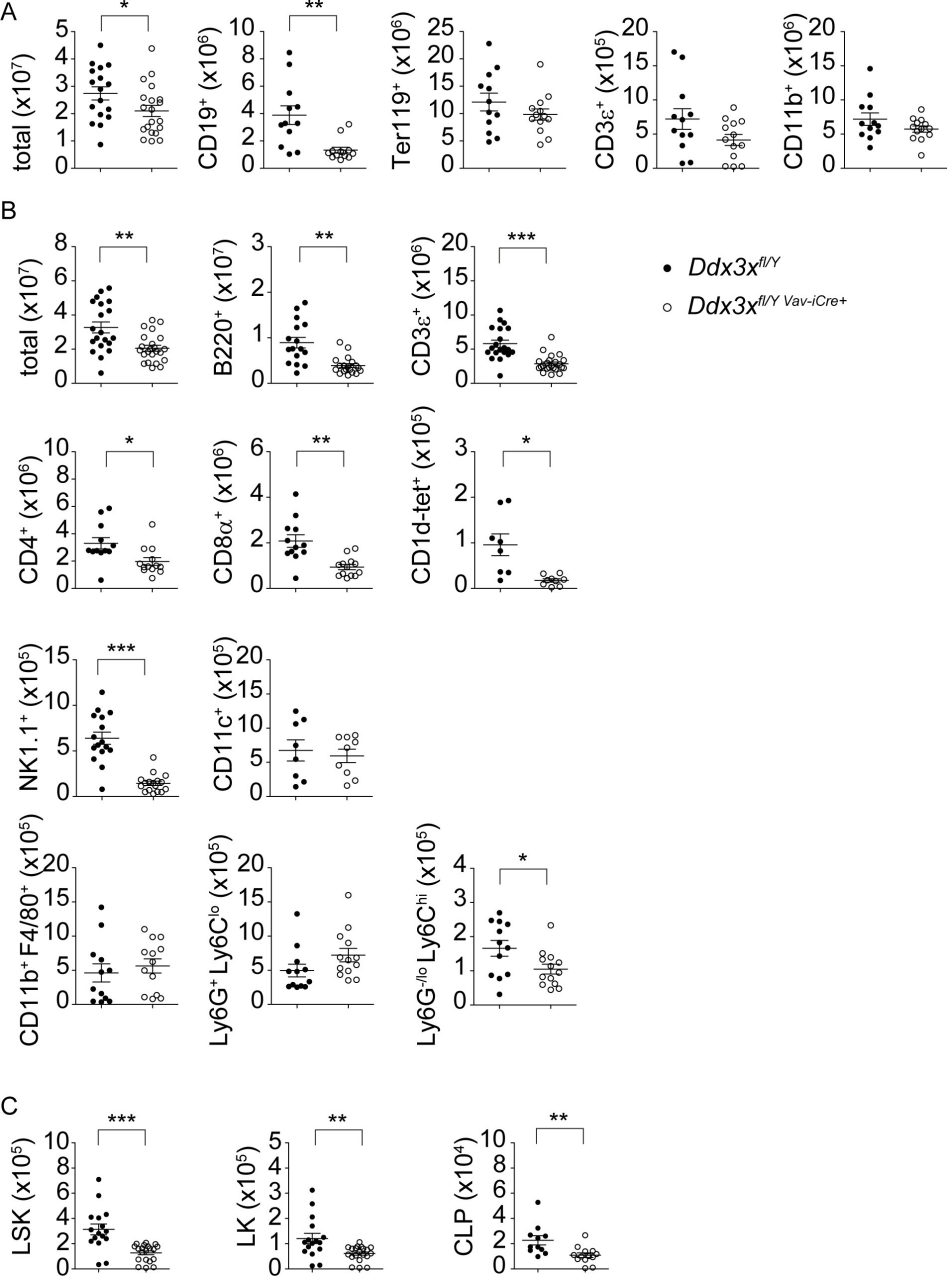
*Ddx3x*^fl/y *Vav-iCre*^ mice show altered cellular compositions in bone marrow and spleen. **A** Bone marrow cells were isolated from uninfected *Ddx3x*^fl/y^ and *Ddx3x*^fl/y *Vav-iCre*^ mice and stained for indicated surface markers. Total cell numbers (referring to one femur and one tibia) are shown. n = 17 *Ddx3x*^*fl/y*^ and 20 *Ddx3x*^*fl/y Vav-iCre*^ mice for total, analyzed in 5 independent experiments. n = 12/13 for CD19^+^, Ter119^+^, CD3^+^ and CD11b^+^, analyzed in 4 independent experiments. **B** Spleens were isolated from uninfected *Ddx3x*^fl/y^ and *Ddx3x*^fl/y *Vav-iCre*^ mice and stained for indicated surface markers. Total cell numbers per organ are shown. *Ddx3x*^fl/y^ (n = 20) and *Ddx3x*^fl/y *Vav-iCre*^ mice (n = 23) were analyzed for total and CD3^+^ cell counts in 6 independent experiments. B220^+^ cells were analyzed in 5 independent experiments (n = 16/19). CD4^+^ and CD8^+^ (both pre-gated on CD3^+^), CD11b^+^F4/80^+^ and Ly6G^+^Ly6C^lo^ and Ly6C^hi^Ly6G^-/lo^ (both pre-gated on CD11b^+^), were analyzed in 4 independent experiments (n = 12/13). NK1.1^+^ cells (pre-gated on CD3^-^), were analyzed in 4 independent experiments (n = 16/17). CD1d-tetramer^+^ (pre-gated on CD3^+^) and CD11c^+^cells were analyzed in 3 independent experiments (n = 8/9). **C** Bone marrow cells were isolated from uninfected *Ddx3x*^fl/y^ and *Ddx3x*^fl/y *Vav-iCre*^ mice and stained with a lineage mix (consisting of Gr1, Mac1, Ter119, B220 and CD3) and antibodies against cKit, Sca1 and CD127. Total cell numbers (referring to one femur and one tibia) for lineage^-^ Sca1^+^ cKit^+^ (LSK), lineage^-^ Sca1^-^ cKit^+^ (LK), as well as lineage^-^ Sca1/cKit^double-dim^ CD127^+^ common lymphoid progenitor (CLP) cells are shown (n = 16 for *Ddx3x*^*fl/y*^ and 20 for *Ddx3x*^*fl/y Vav-iCre*^ mice for LSK and LK cells, analyzed in 4 independent experiments; n = 11/14 for CLP cells, analyzed in 4 independent experiments).

To address the consequences of DDX3X deficiency and to seek an explanation for reduced resistance to Lm, we determined the composition of mature hematopoiesis-derived cell populations in bone marrow and spleen. In the bone marrow total cell numbers were slightly reduced due to a rather selective loss of bone marrow B lymphocytes (Fig 3A). We found splenic composition to be more dramatically changed. Besides B220^+^ B cells the numbers of CD3^+^CD4^+^ and CD3^+^CD8^+^ T cells, CD3^+^CD1d-tetramer^+^ NKT cells and CD3^-^NK1.1^+^ NK cells were significantly reduced in *Ddx3x*^*fl/y Vav-iCre*^ mice. Interestingly, cells of myeloid origin were not generally affected. Numbers of total CD11c^+^ dendritic cells, CD11b^+^Ly6G^+^Ly6C^lo^ neutrophils and CD11b^+^F4/80^+^ macrophages unaltered and only CD11b^+^Ly6C^hi^ monocytes showed a slight reduction (Fig 3B).

**Fig 4:**
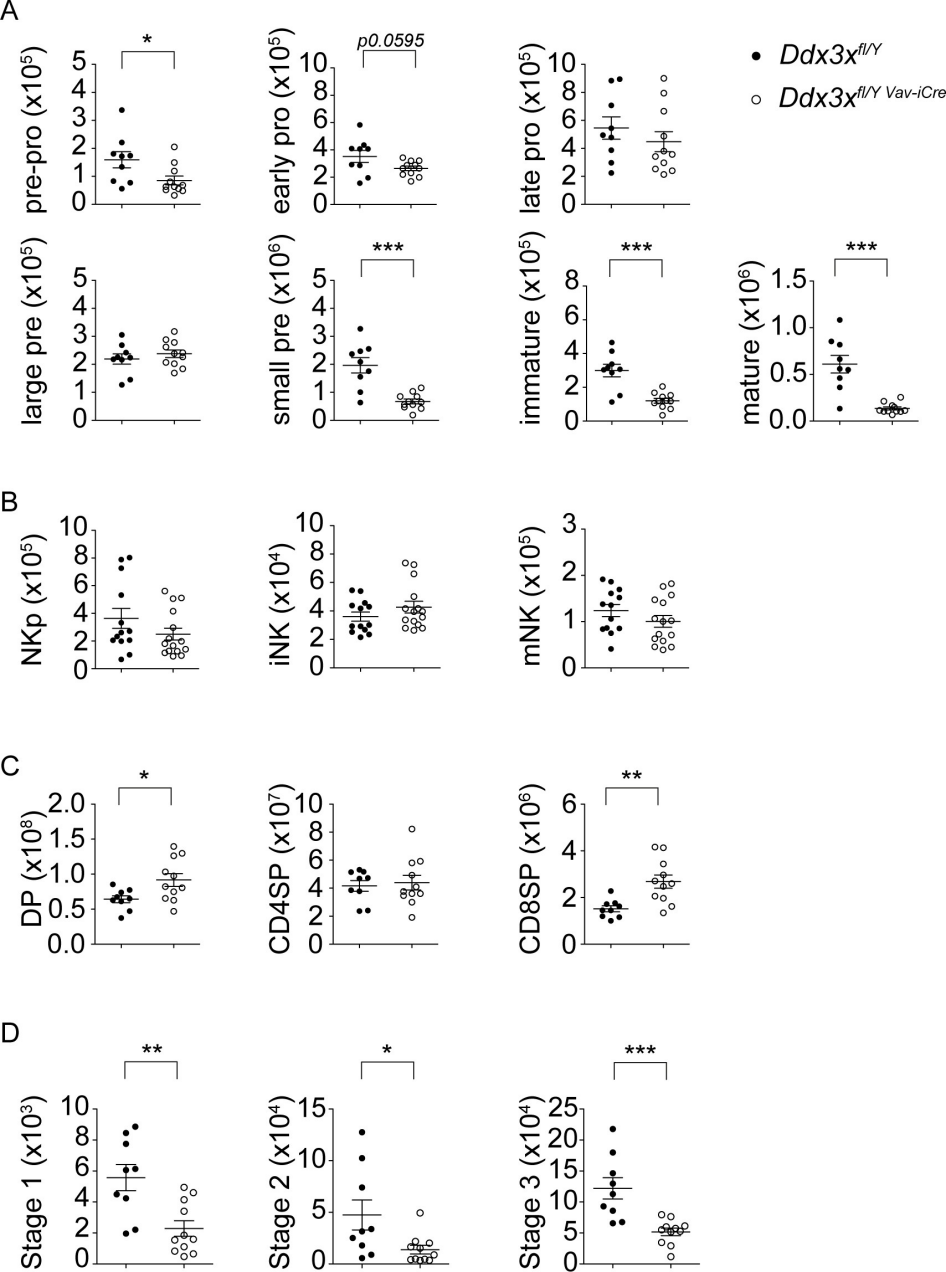
Loss of DDX3X affects hematopoiesis. **A** Bone marrow cells were isolated from uninfected *Ddx3x*^fl/y^ and *Ddx3x*^fl/y *Vav-iCre*^ mice and stained for B220, CD43, BP1, CD24 and IgM. Total cell numbers (referring to one femur and one tibia) of CD43^+^ B220^+^ CD24^-^ BP1^-^ pre-pro B cells, CD43^+^ B220^+^ CD24^+^ BP1^-^ early pro B cells, CD43^+^ B220^+^ CD24^lo^ BP1^+^ late pro B cells, CD43^+^ B220^+^ CD24^hi^ BP1^+^ large pre B cells, CD43^-^ B220^+^ small pre B cells, B220^lo^ IgM^+^ immature B cells and B220^hi^ IgM^+^ mature B cells are shown (n = 9 for *Ddx3x*^fl/y^ and 11 for *Ddx3x*^fl/y *Vav-iCre*^ mice, analyzed in 2 independent experiments). **B** Bone marrow cells were isolated from uninfected *Ddx3x*^fl/y^ and *Ddx3x*^fl/y *Vav-iCre*^ mice and stained with a lineage mix (consisting of Gr1, Ter119, B220 and CD3) and antibodies against CD122, NK1.1 and Dx5. Total cell numbers (referring to one femur and one tibia) of lineage^-^ CD122^+^ NK1.1^-^ Dx5^-^ NK progenitors (NKp), lineage^-^ CD122^+^ NK1.1^+^ immature NK cells (iNK) and lineage^-^ CD122^+^ NK1.1+ Dx5+ mature NK cells (mNK) are shown (n = 13 for *Ddx3x*^fl/y^ and 15 for *Ddx3x*^fl/y *Vav-iCre*^ mice, analyzed in 3 independent experiments).**C** Thymi were isolated from uninfected *Ddx3x*^fl/y^ and *Ddx3x*^fl/y *Vav-iCre*^ mice and stained for CD4 and CD8. Total cell numbers per organ of CD4^+^ CD8^+^ (DP), CD4^+^ CD8^-^ (CD4SP) and CD4^-^ CD8^+^ (CD8SP) are shown (n = 9 for *Ddx3x*^fl/y^ and 11 for *Ddx3x*^fl/y *Vav-iCre*^ mice, analyzed in 2 independent experiments). **D** Thymi were isolated from uninfected *Ddx3x*^fl/y^ and *Ddx3x*^fl/y *Vav-iCre*^ mice and stained with PBS-57 loaded CD1d-tetramer as well as antibodies against CD24, CD44 and NK1.1. Total numbers per organ of CD1d-tet^+^ CD24^-^ CD44^-^ NK1.1^-^ (stage 1), CD1d-tet^+^ CD24^-^ CD44^+^ NK1.1^-^ (stage 2) and CD1d-tet^+^ CD24^-^ CD44^+^ NK1.1^+^ (stage 3) NKT cells are shown (n = 9 *Ddx3x*^fl/y^ and 11 *Ddx3x*^fl/y *Vav-iCre*^ mice, analyzed in 2 independent experiments).

To assess whether reduced lymphoid cell numbers were due to alterations in hematopoiesis we counted the numbers of lineage (Gr1, Mac1, Ter119, B220, CD3)—Sca1^+^ cKit^+^ (LSK), lineage^-^ Sca1^-^ cKit^+^ (LK), as well as lineage^-^ Sca1/cKit^double-dim^ CD127^+^ common lymphoid progenitor (CLP) cells and found all three populations to be significantly reduced (Fig 3C), indicating a role for DDX3X in early hematopoietic development. In line with this observation, further analysis of bone marrow B cell development showed a slight reduction in the numbers of pre-pro- and early pro B cells, as well as a particularly strong reduction of progenitors from the small pre- B cell stage onwards, i.e. following the proliferation of large pre B cells (Fig 4A). Surprisingly, the development of NK cell precursors in the bone marrow seemed unaffected by loss of DDX3X, with lineage (Gr1, Ter119, B220, CD3)^-^ CD122^+^ NK1.1^-^ Dx5^-^ NK progenitors (NKp), lineage^-^ CD122^+^ NK1.1^+^ immature NK cells (iNK) and lineage^-^ CD122^+^ NK1.1^+^ Dx5^+^ mature NK cells (mNK) all present at normal numbers, indicating that the reduction in CLP cells can be compensated for during NK cell development (Fig 4B).

Since the numbers of splenic T and NKT cells were also dramatically affected in *Ddx3x*^*fl/y Vav-iCre*^ mice we more closely examined development of these lineages in the thymus. Interestingly, the number of thymic CD4/CD8 double-positive (DP) and CD8 single-positive (SP) thymocytes was increased in absence of DDX3X, while CD4 SP thymocytes seemed unaffected, suggesting that a developmental block is not responsible for the reduced numbers of mature T cells observed in the periphery (Fig 4C). In contrast, NKT cell development in the thymus was more dramatically affected with CD1d-tetramer^+^ CD24^-^ stage 1, CD1d-tetramer^+^ CD24^-^ CD44^+^ stage 2 and CD1d-tetramer^+^ CD24^-^ CD44^+^ NK1.1^+^ stage 3 NKT cells all strongly reduced in numbers (Fig 4D).

In summary, our analysis of hematopoiesis in *Ddx3x*^*fl/y Vav-iCre*^ mice indicated a differential impact on distinct lineages. While the reduced numbers of peripheral B and NKT cells can be at least partially explained by developmental alterations, the lack of splenic T and NK cells does not seem to result from an obvious developmental block.

**Fig 5:**
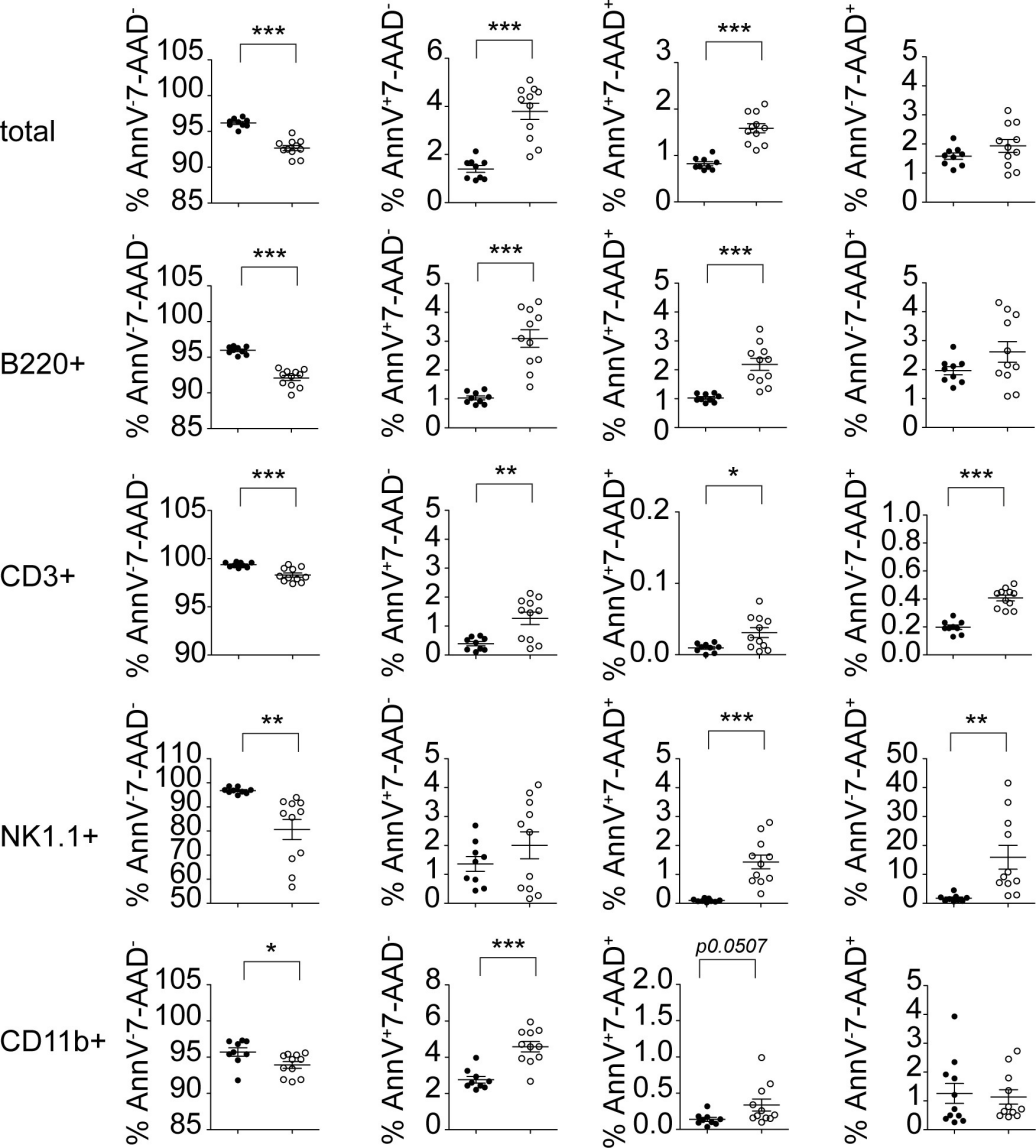
Absence of DDX3X increases hematopoietic cell death. Spleens were isolated from uninfected *Ddx3x*^fl/y^ and *Ddx3x*^fl/y *Vav-iCre*^ mice and stained with fluorochrome-coupled Annexin V plus the indicated surface markers, followed by staining with 7-AAD. Percentage of Annexin V^+^ and/or 7AAD^+^ cells are shown, as indicated (n = 9 for *Ddx3x*^fl/y^ and 11 for *Ddx3x*^fl/y *Vav-iCre*^ mice, analyzed in 2 independent experiments). Statistical analysis was carried out using unpaired, two-tailed Student's *t*-test. Mean and standard error of the mean (SEM) are indicated in the graphs. *P<0.05, **P<0.01, ***P<0.005.

Given that DDX3X-deficient embryos show increased apoptosis we investigated whether cell death might contribute to the reduction of DDX3X-deficient splenic leukocytes. As shown in Fig 5, the absence of DDX3X caused elevated rates of cell death in B220^+^ B cells, CD3^+^ T cells and CD3^-^ NK1.1^+^ NK cells, and to a lesser extent in CD11b^+^ myeloid cells. These results suggest that DDX3X, besides its variable roles in the development of different immune cell lineages, also plays a role in the maintenance of immune cells in the periphery, with the most profound effects observed in lymphocytes and splenic NK cells.

**Fig 6:**
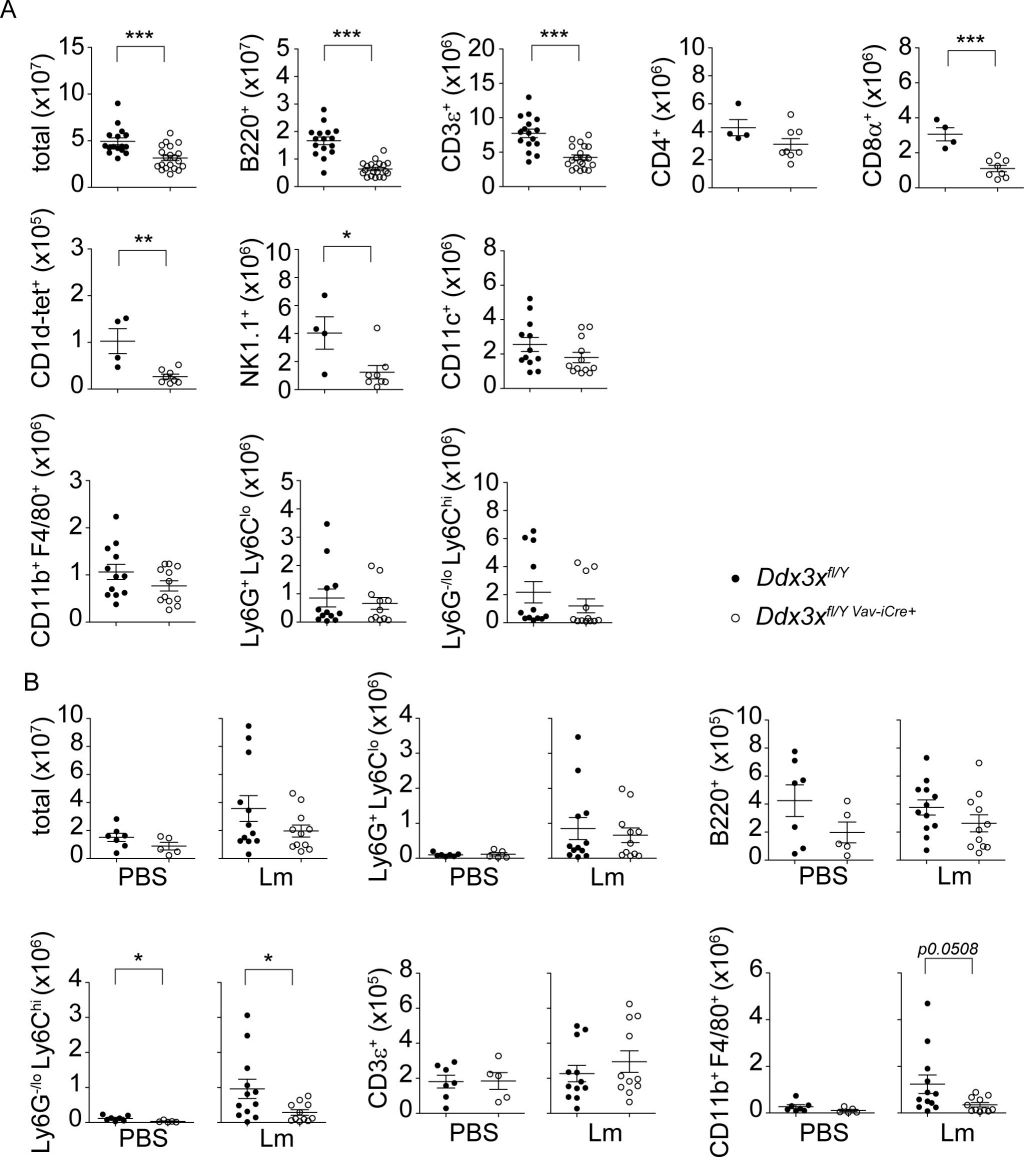
*Ddx3x*^fl/y *Vav-iCre*^ mice show alterations in spleen and peritoneum upon *Listeria monocytogenes* infection. **A** *Ddx3x*^fl/y^ and *Ddx3x*^fl/y *Vav-iCre*^ mice were infected intraperitoneally with 1x10^5^ CFU *Listeria monocytogenes* (strain EGD) for 24 h, spleens were isolated, homogenized and stained with the indicated surface markers, followed by flow cytometry. Total cell numbers per organ are shown (n = 16 for *Ddx3x*^fl/y^ and 20 for *Ddx3x*^fl/y *Vav-iCre*^ mice for total, B220^+^ and CD3^+^ cells, analyzed in 5 independent experiments; n = 4/8 for CD4^+^, CD8^+^ and CD1d-tetramer^+^ cells, all pre-gated on CD3^+^, analyzed in 2 independent experiments; n = 4/8 for NK1.1^+^ cells (pre-gated on CD3^-^), analyzed in two independent experiments; n = 12/12 for CD11c^+^, CD11b^+^F4/80^+^ cells, analyzed in 3 independent experiments; n = 12/12 for Ly6G^+^Ly6C^-/lo^ and Ly6G^lo^Ly6C^hi^ cell populations, both pre-gated on CD11b^+^and analyzed in 3 independent experiments). **B** *Ddx3x*^fl/y^ mice and *Ddx3x*^fl/y *Vav-iCre*^ mice were infected intraperitoneally with 1x10^5^ CFU *L*. *monocytogenes* (strain EGD) for 24h or treated with PBS as a control. Peritoneal exudate cells were collected via peritoneal lavage and stained with the indicated surface markers. Total cell numbers of B220^+^ B cells, CD3^+^ T cells, Ly6G^+^Ly6C^lo^ neutrophils and Ly6G^-/lo^ Ly6C^hi^ monocytes (both pre-gated on CD11b^+^) and CD11b^+^ F4/80^+^ macrophages are shown. n = 7 (*Ddx3x*^fl/y^ mice) and 5 (*Ddx3x*^fl/y *Vav-iCre*^ mice) for PBS controls and n = 12/11 for infected animals. P-values were calculated using the unpaired, two-tailed Student's *t*-test. Mean and standard error of the mean (SEM) are indicated in the graphs. *P<0.05, **P<0.01, ***P<0.005.

To determine whether Lm infection exacerbates defects in hematopoiesis, splenic cell populations were analysed in infected mice. 24 hours of infection increased the number of total splenocytes in both control and *Ddx3x*^*fl/y Vav-iCre*^ mice (first panels of Figs 3A and 6A, Table 1), mainly due to recruitment of Ly6C^hi^ inflammatory monocytes (approx.13-fold versus 12-fold mean increase, respectively), suggesting no obvious recruitment defects caused by the absence of DDX3X. By and large, the cell populations suffering from DDX3X deficiency were the same as in uninfected animals with B220^+^ B cells, CD3^+^ CD8^+^ T cells, CD3^+^ CD1d-tetramer^+^ NKT cells and CD3^-^ NK1.1^+^ NK cells being particularly compromised. By comparison, DDX3X-deficient myeloid cells showed a rather mild decrease that did not reach statistical significance (Fig 6A). Table 1 summarizes the infection-induced relative changes in splenic leukocyte composition. While some differences in the recruitment and/or proliferation of different leukocyte populations was observed, this display of the data emphasizes that most changes in leukocyte numbers precede infection rather than being result of the innate response to Lm.

**Table 1:** Changes in spleen cell populations after 24h of infection with *Listeria monocytogenes*. Numbers in columns 2 and 4 represent relative changes of the indicated cell populations in *Ddx3x*^fl/y^ and *Ddx3x*^fl/y *Vav-iCre*^ mice, infected for 24h with *L*. *monocytogenes* (Lm). Numbers in column 6 show differences in cell population changes between the two genotypes, expressed as the ratio *Ddx3x*^fl/y *Vav-iCre*^*/Ddx3x*^fl/y^. P values in columns 3 and 5 indicate whether any changes between uninfected and infected mice reached statistical significance; *P<0.05, **P<0.01.

	*Ddx3x*^*fl/Y*^ 24h Lm /uninfected (fold change)	*p*	*Ddx3x*^*fl/Y Vav-iCre+*^ 24h Lm/uninfected (fold change)	*p*	*Ddx3x*^*fl/Y Vav-iCre*^ */Ddx3x*^*fl/Y*^ (ratio fold change)
total	1.51	**	1.53	**	1.01
B220^+^	1.55	**	1.64	**	1.06
CD3^+^	1.17	*	1.44	**	1.23
CD4^+^	1.12	n.s.	1.58	*	1.41
CD8^+^	1.24	n.s.	1.18	n.s.	0.95
NK1.1^+^	0.59	n.s.	0.74	n.s.	1.26
CD1d-tet^+^	0.80	n.s.	1.52	n.s.	1.90
CD11b^+^ F4/80^+^	2.12	**	1.36	n.s.	0.64
Ly6G^+^ Ly6C^lo^	1.72	n.s.	0.91	n.s.	0.53
Ly6G^lo^ Ly6C^hi^	13.12	*	11.50	*	0.88
CD11c^+^	1.51	*	1.51	n.s.	1.00

In our animal experiments, the primary site of Lm infection is the peritoneal cavity. We therefore analysed cell recruitment to this anatomical location. The data summarized in Fig 6B show that unlike the spleen hematopoietic DDX3X deficiency per se has little effect on immune cells residing in the peritoneum, with only the number of Ly6C^hi^ monocytes slightly reduced in PBS treated control animals. Upon infection with Lm we observed a rise in peritoneal exudate cell (PEC) numbers owing primarily to recruitment of Ly6C^lo^Ly6G^+^ neutrophils, Ly6C^hi^ monocytes and CD11b^+^F4/80^+^ macrophages. While neutrophil recruitment seemed unaffected, the number of inflammatory monocytes was significantly decreased in *Ddx3x*^*fl/y Vav-iCre*^ animals and we observed a tendency for lower macrophage numbers. These results indicate a requirement for DDX3X in the generation, maintenance or recruitment of inflammatory monocytes particularly in the peritoneal cavity.

### Mice lacking DDX3X in the hematopoietic system produce reduced amounts of serum IL-12 and IFNγ after *L*. *monocytogenes* infection

**Fig 7:**
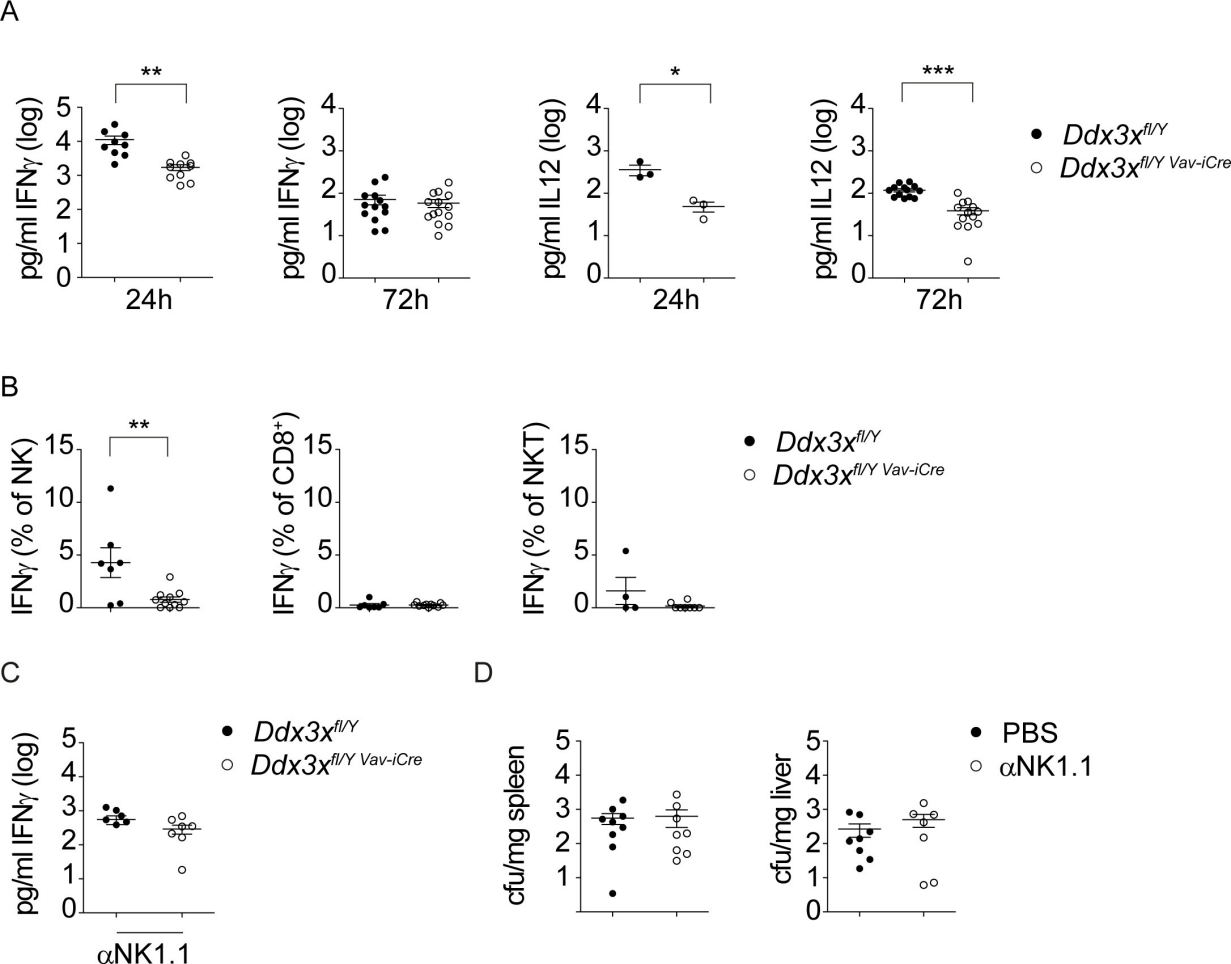
Reduced NK-mediated IFNγ production in *Ddx3x*^fl/y *Vav-iCre*^ mice infected with *Listeria monocytogenes*. **A** *Ddx3x*^fl/y^ and *Ddx3x*^fl/y *Vav-iCre*^ mice were infected intraperitoneally with 1x10^5^ CFU *L*. *monocytogenes* (strain EGD) for the indicated periods of time. n ≥ 3 for each genotype. Mice were sacrificed and serum was collected. Cytokine levels were analyzed by flow cytometry-based bead array. **B** *Ddx3x*^fl/y^ and *Ddx3x*^fl/y *Vav-iCre*^ mice were infected intraperitoneally with 1x10^5^ CFU *L*. *monocytogenes* (strain EGD) for 24 h, spleens were isolated, homogenized and splenocytes were stained for indicated surface markers followed by fixation, permeabilization and intracellular staining for IFNγ. Data show percent of IFNγ-producing CD3ε^-^NK1.1^+^NKp46^+^ (NK), CD3ε^+^CD8^+^ (CD8^+^) and CD3ε^+^CD1d-tet^+^ (iNKT) cells. n = 7 for *Ddx3x*^fl/y^ and n = 11 for *Ddx3x*^fl/y *Vav-iCre*^ mice, analyzed in 3 independent experiments. **C** After depleting NK-cells with an anti-NK1.1 antibody, *Ddx3x*^fl/y^ and *Ddx3x*^fl/y *Vav-iCre*^ mice were infected intraperitoneally with 1x 10^5^ CFU *L*. *monocytogenes* (strain EGD) for 24 h. Mice were sacrificed, serum was collected and IFNγ levels were determined by ELISA. n = 6 for *Ddx3x*^fl/y^ and 7 for *Ddx3x*^fl/y *Vav-iCre*^ mice, analyzed in 2 independent experiments. **D** After depleting NK-cells with an anti-NK1.1 antibody, wt mice were infected intraperitoneally with 1x 10^5^ CFU *L*. *monocytogenes* (strain EGD) for 72 h. The mice were sacrificed and the bacterial load was determined in spleen and liver by CFU assay. Lines in all panels represent the mean with standard error of the mean (SEM). Statistical significance was calculated using the unpaired, two-tailed Student's *t*-test. *P<0.05, **P<0.01, ***P<0.005.

To address potential protective immune mechanisms under DDX3X control we determined the levels of several serum cytokines and chemokines in mice infected with Lm. Most of the factors we assessed were not significantly different between *Ddx3x*^*fl/y Vav-iCre*^ mice and control animals. At 24 hours after infection, serum levels of Il-6, IL-17, IL-10, TNFα and IL1β were unchanged and while the levels of Il-6, IL-10, TNFα and IL1β increased in *Ddx3x*^*fl/y Vav-iCre*^ relative to control animals at 72h, the difference only reached significance in the case of IL-10. In addition, we observed a small but significant reduction in IL-4 levels at both time points as well as reduced CCL5/RANTES concentrations at 24 hours (S1 Fig). The most striking difference was the reduction of serum IFNγ and IL-12 in *Ddx3x*^*fl/y Vav-iCre*^ mice at 24 hours after infection. At 72hrs IFNγ levels of *Ddx3x*^*fl/y Vav-iCre*^and control mice were similar, either due to a recovery of the former, or to more rapid subsiding of IFNγ production in the latter animals (Fig 7A). The results suggest an inhibited IL12-IFNγ axis in the early stage of Lm infection of *Ddx3x*^*fl/y Vav-iCre*^ mice.

Upon *L*. *monocytogenes* infection, early production of IFNγ by NK-cells, NKT cells and CD8^+^ T-cells is crucial for the activation of macrophage effector functions and subsequent bacterial clearance [43–45]. Early IFNγ production requires IL-12, as IL-12 depletion leads to abrogated IFNγ levels and reduced resistance to infection [36,38,46,47]. To address the contribution of these cell populations to the differences seen in IFNγ levels 24 hours post-infection, flow-cytometric analysis of IFNγ production was performed. We found that NK cells were an important source of IFNγ in wt animals at this early time point, because the fraction of producer cells was the largest among the investigated cell types (Fig 7B). Numbers of NK cells are much lower in *Ddx3x*^*fl/y Vav-iCre*^ mice and of the few remaining cells a smaller fraction produced IFNγ, possibly as a consequence of reduced IL-12 amounts. The fraction of IFNγ-producing splenic iNKT cells was low and not significantly affected by genotype, while IFNγ– producing CD8^+^ T cells were below the limit of detection (Fig 7B). These data suggest that NK cells make an important contribution to serum IFNγ in the early phase of Lm infection, and their absence in *Ddx3x*^*fl/y Vav-iCre*^ mice is largely responsible for reduced serum levels of IFNγ at the 24 hour time point.

To verify our assumption that NK cells are the primary producers of IFNγ at 24 hours after Lm infection, we depleted NK cells from *Ddx3x*^*fl/y*^ and *Ddx3x*^*fl/y Vav-iCre*^ mice using an anti-NK1.1 antibody. 72 hours after intraperitoneal administration about 98% of NK cells were depleted, while splenic CD1d-tet^+^ iNKT cells remained unaffected under these conditions. The NK-depleted mice were then infected with Lm and 24 hours later serum was collected. Depletion of NK-cells strongly reduced serum IFNγ from about 10^4^pg/ml to less than 10^3^pg/ml after NK depletion (leftmost panel in Fig 7A and 7C). NK depletion also abolished the differences between wild type controls and *Ddx3x*^*fl/y Vav-iCre*^ mice (Fig 7C). In line with earlier findings [48] [49], depletion of NK cells did not reduce the bacterial burden in infected organs of wt mice (Fig 7D). Therefore, although our data emphasize the contribution of NK cells to serum IFNγ production during the early, innate immune response against Lm, these results suggest that residual serum IFNγ in NK-depleted mice, or local production by other cells, suffices for macrophage activation in the early phase of Lm infection. Our data also support our notion that NK cells are the primary cell type responsible for the lack of sufficient early serum IFNγ production in mice lacking hematopoietic DDX3X.

### DDX3X-deficient macrophages show impaired inflammatory cytokine production and are unable to control *L*. *monocytogenes* growth

**Fig 8:**
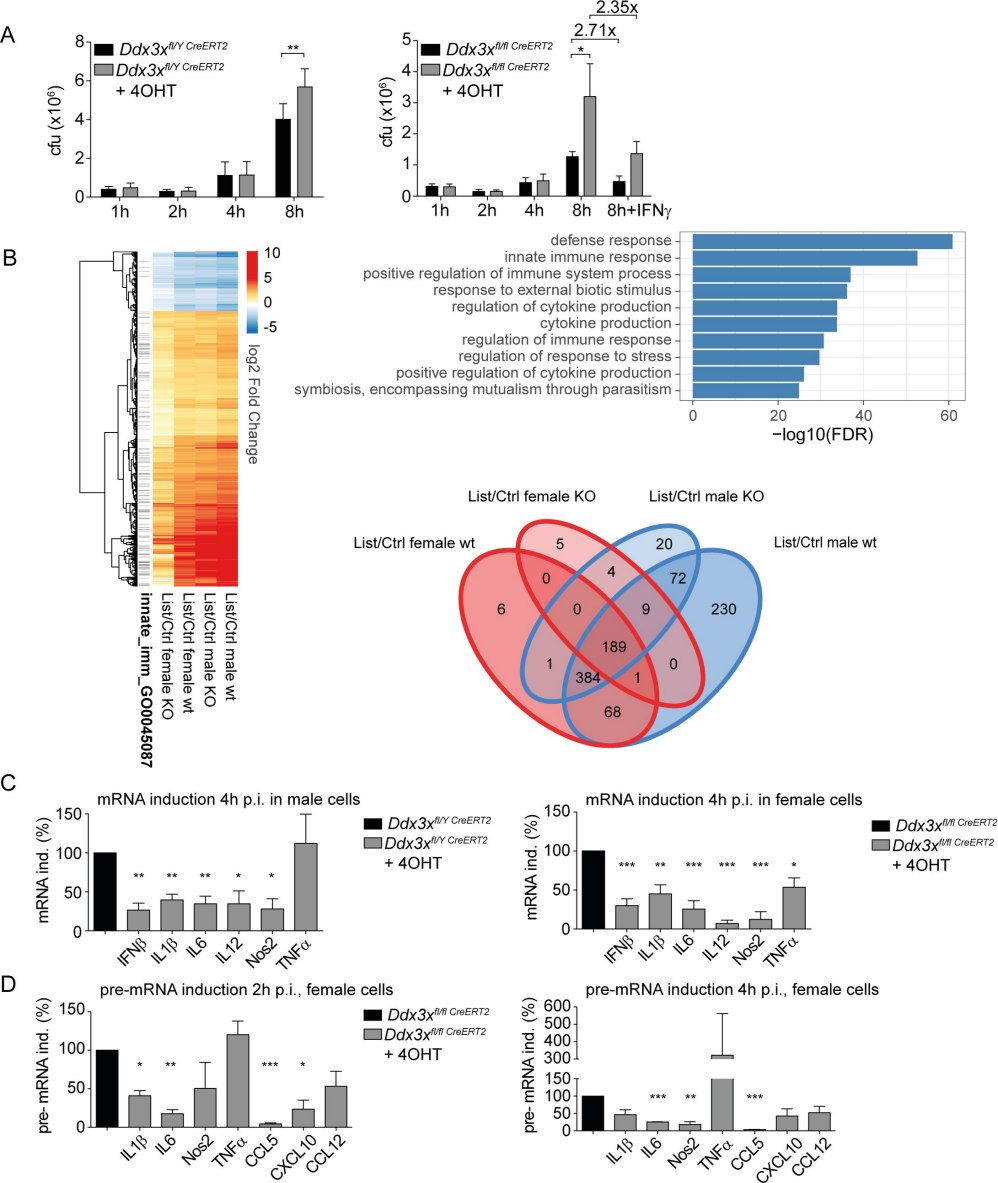
Loss of DDX3X compromises the immune response of macrophages to *Listeria monocytogenes*. **A** Bone marrow-derived macrophages (BMDMs) of *Ddx3x*^fl/y Cre*ERT2*^ male and *Ddx3x*^fl/fl Cre*ERT2*^ female mice were infected with *L*. *monocytogenes* (strain LO28) with a multiplicity of infection (MOI) of 10. Colony-forming units (CFU) were determined 1, 2, 4 or 8 h after infection by plating serial dilutions of lysed cells on brain–heart infusion (BHI) agar plates. The last two columns in the right panel represent cells pretreated with IFNγ over night. n = 6, analyzed in 2 independent experiments, for male BMDMs (left panel). n = 12, analyzed in 4 independent experiments, for female BMDMs (right panel). Data are represented as mean values +/- standard deviations (SD). Statistical analysis was performed using the unpaired, two-tailed Student's *t*-test. * P<0.05. **B** BMDM of mice with the indicated genotypes were left untreated or infected with *L*. *monocytogenes* at MOI 10 for 4 hours as described below. RNA isolated from the infected cells was isolated and subjected to RNA-Seq. Left panel: heat map of log 2-fold gene expression changes between Listeria-treated and untreated samples for genes differentially expressed upon treatment in WT samples of either sex (padj <0.01, abs(logFC)>1; DeSeq2 v1.18.1). Genes assigned to the GO category GO:0045087 innate immune response are indicated on the left. From left to right, genes from female knock-out, female wild-type, male knock-out and male wild-type cells (as indicated below the lanes) are compared. Upper right panel: functional enrichment analysis of the differentially expressed genes using DAVID v6.8; the top 10-enriched GO terms ranked by p-value for the category GOTERM_BP_3 are shown. Lower right panel: Venn diagram of gene sets differentially expressed upon Lm infection in the four experimental conditions displayed in the heat map (DESeq2 v 1.18.1; absolute log2 fold change > 1; FDR < 0.01). Solid blue: Lm-infected versus uninfected in macrophages representing a male wildtype genotype (DDX3X^fl/y^); light blue: Lm-infected versus uninfected in macrophages representing a male DDX3X-deficient genotype (DDX3X^-/y^); Solid red: Lm-infected versus uninfected in macrophages representing a female wildtype genotype (DDX3X^fl/fl^); light red: Lm-infected versus uninfected in macrophages representing a female DDX3X-deficient genotype (DDX3X^-/-^). **C** Bone marrow-derived macrophages (BMDMs) of *Ddx3x*^fl/y Cre*ERT2*^ male and *Ddx3x*^fl/fl Cre*ERT2*^ female mice with or without 4_OHT-mediated DDX3X deletion, were infected with *L*. *monocytogenes* (strain LO28) with a multiplicity of infection (MOI) of 10. After 4 h of infection total RNA was isolated and analyzed by qPCR for expression of the indicated genes. Upper panels show results from female cells, lower panels show results from male cells, normalized to their respective controls (male and female macrophages without 4-OHT treatment = 100%). Bars represent mean values +/- standard deviations (SD) of at least three independent experiments. Statistical significance was calculated using the paired, two-tailed Student's *t*-test. *P<0.05, **P<0.01, ***P<0.005. **D** Primers flanking intron sequences were chosen to analyze the primary unprocessed transcripts in untreated or 4-OHT treated BMDMs of *Ddx3x*^fl/fl Cre*ERT2*^ female mice. Cells were infected with *L*. *monocytogenes* (strain LO28) with a multiplicity of infection (MOI) of 10. After 2 and 4 h of infection total RNA was isolated and analyzed by qPCR for expression of the indicated primary transcripts. Bars represent mean values +/- standard deviations (SD) of at least three independent experiments, normalized to male and female controls (macrophages without 4-OHT treatment = 100%). Statistical significance was calculated using the paired, two-tailed Student's *t*-test. *P<0.05, **P<0.01, ***P<0.005.

Macrophages are essential effector cells against Lm. Reduced proinflammatory activity and/or cell-autonomous antimicrobial states in absence of DDX3X might thus explain the reduced innate immunity of *Ddx3x*^*fl/y Vav-iCre*^ mice. To study antimicrobial responses of both male and female sexes we generated bone marrow derived macrophages (BMDMs) from *Ddx3x*^*fl/fl CreERT2*^ and *Ddx3x*^*fl/y CreERT2*^ mice. *In vitro* deletion of DDX3X by tamoxifen treatment allowed us to address the effect of *Ddx3x* deletion in the presence or absence of the *Ddx3y* gene. Tamoxifen treatment after 5 days of culturing bone marrow cells in CSF-1-containing medium did not impair the terminal differentiation of macrophages from *Ddx3x*^*fl/fl CreERT2*^ female and *Ddx3x*^*fl/y;CreERT2*^ male mice. However, the fully differentiated, female *Ddx3x*^*-/-*^ cells showed a reduced life span and were used no later than 3 days after the termination of tamoxifen treatment. To determine the impact of DDX3X on cell-autonomous clearance of Lm, BMDMs of male (Fig 8A, left panel) and female mice (Fig 8A, right panel) were infected with Lm at an MOI of 10 and colony forming unit (CFU) assays were performed to monitor bacterial growth. DDX3X-deficient cells from both sexes contained higher bacterial loads compared to the wild-type cells 8 hours after infection. Activation of DDX3X-deficient female cells with IFNγ resulted in increased killing of intracellular Lm. The relative drop in bacterial loads were similar to wt (2.3 versus 2.7-fold) but in absolute numbers about twice as many bacteria persisted in DDX3X-deficient macrophages (Fig 8A, left panel). The data suggest that IFNγ responsiveness per se is unaffected, but that the remaining difference in bacterial numbers may result from a higher initial burden compared to wt cells.

The consequences of DDX3X deficiency on the expression of genes contributing to the innate antimicrobial response of macrophages were examined by RNA-Seq analysis of untreated or Lm-infected cells. Genes differentially expressed upon Lm-infection in either male or female cells (S1 Table) are enriched for GO Immune System terms, and the extent to which DDX3X deficiency altered the transcriptome depended on the sex chromosomes of the cells (Fig 8B, heatmap and upper right panel). This suggests that one or more Y-chromosomal genes partially compensate for the loss of DDX3X at the level of transcript synthesis. The data demonstrate control of the macrophage transcriptome by DDX3 isoforms and, in line with the mouse phenotypes, an incomplete redundancy between DDX3X and DDX3Y and/or additional genes present on the Y chromosome.

wt mice of female sex show higher susceptibility to Lm infection and immune-related genes may show sex-dependent differences in expression [50]. In line with this, we observed a considerable variation in the differentially expressed genes in macrophages of infected wt male and wt female mice (heatmap of Fig 8B). Comparison of gene sets differentially expressed upon Lm infection at the same significance threshold in the four experimental conditions revealed that a set of 311 genes responded with altered expression in wt males, but not in wt females, whereas only 7 genes responded to infection in females, but not in males (Fig 8B, Venn diagram). 230 of the 311 male-specific genes and all of the 7 female-specific genes were unresponsive to infection in macrophages of the same sex lacking DDX3X. The data suggest a contribution of DDX3X to sex-specific gene expression in response to infection with Lm.

**Fig 9:**
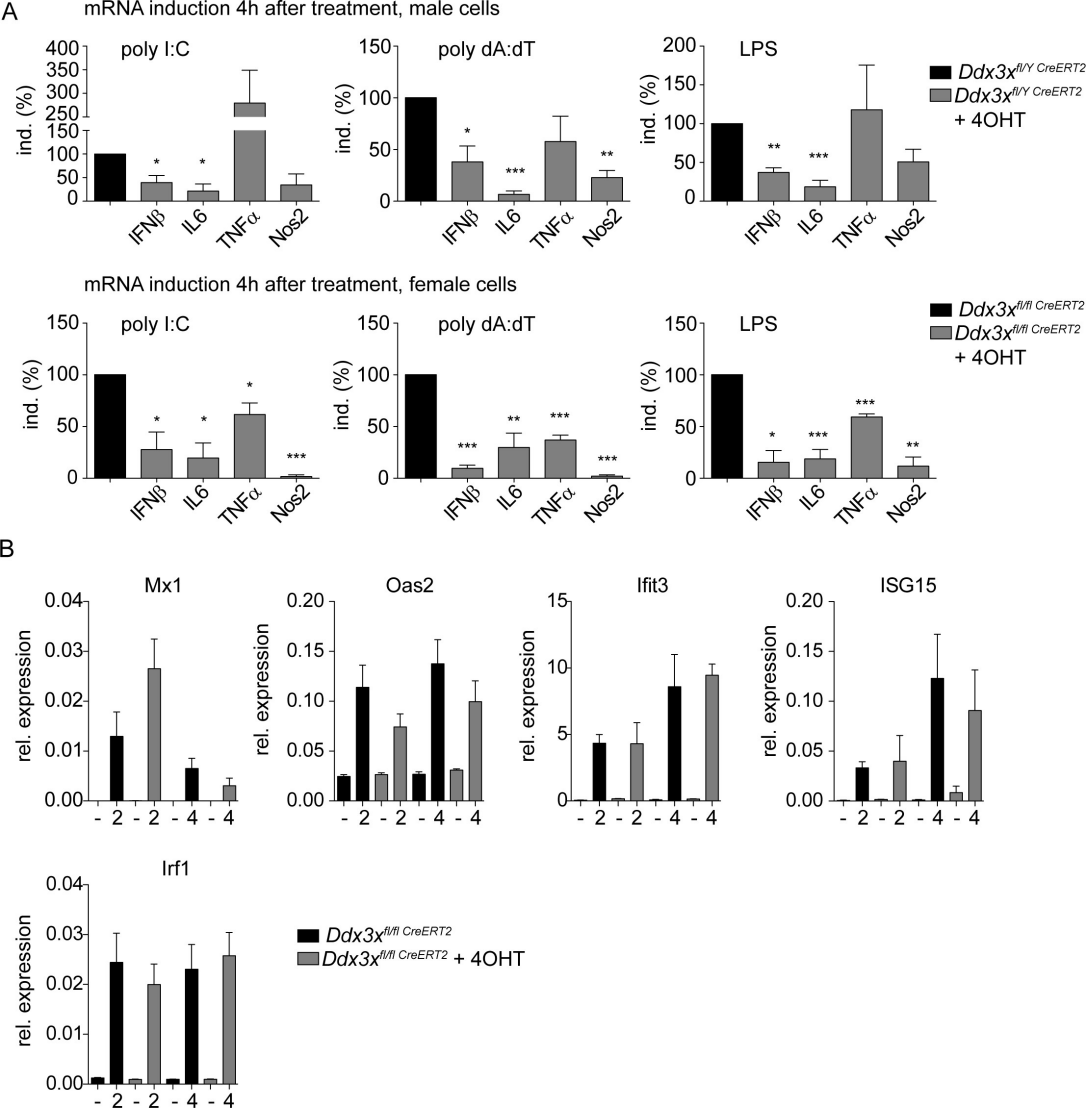
DDX3X deficient macrophages show compromised response to TLR ligands, but respond normally to IFNs. **A** Bone marrow-derived macrophages (BMDMs) of *Ddx3x*^fl/y Cre*ERT2*^ male and *Ddx3x*^fl/fl;Cre*ERT2*^ female mice with or without 4_OHT-mediated DDX3X deletion, were treated with the indicated pathogen-associated molecular patterns (PAMPs). After 4 h of treatment total RNA was isolated and analyzed by qPCR for expression of the indicated genes. Upper panel shows results from male cells, lower panel shows results from female cells. Bars represent mean values +/- standard deviations (SD) of at least three independent experiments, normalized to their respective controls (male and female macrophages without 4-OHT treatment = 100%). Statistical significance was calculated using the paired, two-tailed Student's *t*-test. *P<0.05, **P<0.01, ***P<0.005. **B** Bone marrow-derived macrophages (BMDMs) of *Ddx3x*^fl/fl Cre*ERT2*^ female mice with (grey bars) or without (black bars) 4-OHT-mediated DDX3X deletion, were treated with IFNβ (250U/ml) or IFNγ (5ng/ml) for 2 h or 4 h, as indicated. Total RNA was isolated and expression levels of the indicated genes was analyzed by qPCR. Bars represent mean values +/- standard error of the mean (SEM) of three independent experiments.

Validation of critical antimicrobial genes affected by the lack of the *Ddx3x* gene in RNA-Seq experiments by qPCR is shown in Fig 8C. Cytokines with a strong impact on innate resistance to Lm, such as IL-1, IL-6, IL-12, TNFα as well as chemokines were controlled by DDX3X. Further consistent with the RNA-Seq results, the reduction in expression for most of the examined genes was more pronounced in female (lower panel) than in male macrophages (upper panel). The broad effect of DDX3X deficiency on cytokine production is consistent with the impact of the protein on both IRF3/7 and NFκB pathways (Fig 1C, 1E and 1F). To determine whether the DDX3X effect on these genes is at the level of transcription rather than transcript processing, primary transcript synthesis of a number of mRNAs was examined at 2hrs or 4hrs after infection in female macrophages. Consistent with our earlier observation that DDX3X increases *Ifnb* gene expression at the level of transcription [4], most primary transcripts were reduced, the *Tnfa* transcript being a noteworthy exception (Fig 8D). DDX3X-deficiency also blunted responses to the defined pathogen-associated molecular patterns (PAMPs) poly (I:C), poly (dA:dT) and LPS (Fig 9A). With exception of the *Il6* gene, the impact of DDX3X deficiency on signalling by the respective pattern recognition receptors was more pronounced in female cells (upper versus lower panels). These results show that DDX3X affects both primary transcription as well as transcript processing of genes targeted by microbial sensor molecules. Finally, to assess the production of Interferon stimulated genes (ISGs) in absence of DDX3X we treated DDX3X-ablated and control BMDMs with IFNs. As shown in Fig 9B, the induction of *Mx1*, *Oas2*, *Ifit3* and *Isg15* mRNAs was not significantly altered between genotypes upon stimulation with IFNβ (left panels). Similarly, induction of *Irf1* mRNA after addition of IFNγ was comparable between the genotypes (rightmost panel). These data indicate that DDX3X-deficient macrophages respond normally to exogenous sources of IFN.

## Discussion

In this report we show that DDX3X deficiency in hematopoietic cells results in a striking susceptibility against the bacterial pathogen *Listeria monocytogenes* (Lm). The lack of effect on VSV-infected mice suggests that ablation of DDX3X in the hematopoietic system does not result in a complete breakdown of innate immunity. Immune responses against Lm commence with an early innate phase including macrophages, inflammatory monocytes, NK-cells, DCs, CD4^+^, CD8^+^ and γδT cells that together constrain the infection (Andersson et al., 1998; Lee et al., 2013; Pamer, 2004b; Shi et al., 2011; Unanue, 1997). Protective immunity requires the synthesis of several proinflammatory cytokines such as TNF, IL6 and IL1, whose spatial and temporal appearance is orchestrated by the immune system. The role of IFNγ in the context of Listeriosis is well-established, as evidenced by the increased susceptibility of mice deficient in either IFNγ [37] or the IFNγ receptor (Huang et al., 1993; Lee et al., 2013). Here we identify three factors contributing to the selective susceptibility of mice lacking hematopoietic DDX3X against Lm. First, the selective defects in hematopoiesis reduce the ability to establish the inflammatory milieu needed for innate defence against Lm. Among myeloid cells, inflammatory monocytes are the only cell type strongly dependent on DDX3X and this cell type is indispensable for restricting Lm growth [51]. Second, DDX3X deficiency impairs antimicrobial gene expression in macrophages, a major effector cell against Lm. The absence of the helicase causes a widespread reduction of genes providing innate immunity, amongst which are proinflammatory cytokines, but also intracellular effectors such as iNOS. The findings reported here and by others [5] show that DDX3X enhances the IRF3/7 as well as the NFκB pathways. This provides an explanation for its strong impact on infection-induced genes and poses the question whether other pathways conveying immunity are influenced by DDX3X as well. The alteration of macrophage gene expression has two important consequences, the lack of cell-autonomous defence and, once more, a reduced ability to establish an inflammatory milieu. Third, DDX3X is needed for a fully active IL12-IFNγ axis. Our data support the notion that this results from a defect of IL12 production by macrophages and possibly other cell types, and from a decrease of IFNγ-producing cells.

### Mechanism of DDX3X action, redundancy with DDX3Y

In spite of being described as a substrate of the TBK1 and IKKε kinases we observed that DDX3X enhances the induction of the *Ifnb* gene also in the absence of TBK1/IKKε when a constitutively active phosphomimetic mutant of IRF7 is provided. This demonstrates that DDX3X retains the ability to enhance IRF-mediated transcription in absence of TBK1/IKKε. Therefore, activating phosphorylation in this situation is either dispensable or performed by other kinases, for example, the conventional IKK. The latter interpretation is in line with the ability of DDX3X to enhance NFκB-dependent transcription, but requires experimental confirmation.

*Ddx3x* is essential for development, as its deletion leads to prenatal lethality in mice, where DDX3X is necessary for both embryonic and extraembryonic tissue development [10]. Both DDX3X and DDX3Y are among essential genes in some, but not all investigated human leukemic cell lines [52]. Hematopoietic loss of DDX3X via Vav-Cre mediated deletion resulted in viable male but not female mice, emphasizing the pivotal role of DDX3X in hematopoietic development. The Y-chromosomal homologue DDX3Y shares 90% (mouse) or 92% (human) identity at the protein level. The high degree of homology manifested in its redundant functions in enhancing *Ifnb* gene expression. Therefore, it is tempting to speculate that DDX3Y alone is responsible for the hematopoietic rescue provided by the Y-chromosome. However, this has not been directly tested and a participation of additional Y-chromosomal genes cannot be ruled out. Sofar we have not examined the hematopoietic defect underlying the death of female *Ddx3x*^*fl/y Vav-iCre*^ mice. A failure to produce erythrocytes would seem a likely explanation.

### The role of DDX3X in hematopoietic cells during homeostasis and infection

Analysis of hematopoiesis-derived cells in bone marrow and spleen revealed three degrees of susceptibility to the loss of DDX3X. Particularly B cells were impeded in their development, leading to reduced numbers in both organs. NK cells on the other hand developed normally in the bone marrow, but showed reduced presence in the spleen. This defect may result either from reduced recruitment to the spleen, or a difference in survival conditions between splenic and bone marrow NK cells. Finally, mature myeloid cells such as macrophages, neutrophils, or CD11c+ DC showed little dependence on DDX3X in either organ. One likely explanation for reduced hematopoiesis is an increase in apoptosis in DDX3X-deficient cells, particularly B cells and NK cells. Based on studies in mouse embryos this may result from a p53-mediated response to DNA damage and reflect an activity of DDX3X in cell cycle regulation and/or DNA damage repair (Bol *et al*, 2015; Sun *et al*, 2013; Li *et al*, 2014; Chen *et al*, 2016). In keeping with this notion a preliminary proteomic screen in macrophages identified multiple DDX3X interactors with potential impact on DNA damage and repair pathways. Among these factors are the DBHS family members SFPQ, NONO and PSPC1 [53]. The idea that the increase in apoptosis found in DDX3X-deficient cells is linked to proliferation is supported by cell types found to be particularly sensitive, such as the small preB stage in B cell development, or inflammatory monocytes that, unlike resident splenic macrophages, showed increased apoptosis when lacking DDX3X.

### DDX3X in innate responses to pathogens-potential links to macrophage gene expression

Given the role of DDX3X in the IFN-I synthesis of infected cells we were surprised to find innate immunity to VSV unimpaired. Hematopoietic cells, particularly pDC, are thought to be essential IFN producers during systemic VSV infection [41,42]. One possibility to explain our results is that pDC may not require DDX3X for IFN-I production. Alternatively, serum levels of IFN-I may not accurately reflect the local need to clear infection. This hypothesis is consistent with our findings in Lm-infected mice: NK cells were responsible for high serum IFNγ amounts, but their depletion still left enough IFNγ production for unimpaired innate immunity. An alternative explanation is therefore that our findings with VSV reflect a greater than hitherto suspected need for IFN-I from nonhematopoietic cells. We show that induction of antiviral genes by IFN-I is unimpaired in DDX3X-deficient cells. This further suggests that any IFN-I production defect in *Ddx3x*^*fl/y Vav-iCre*^ mice does not reduce the levels of the cytokines below the threshold needed for the antiviral state.

The susceptibility of *Ddx3x*^*fl/y Vav-iCre*^ mice to *Lm* shows that DDX3Y alone is not generally sufficient to compensate for the lack of DDX3X in the immune system. Due to the lethality of DDX3X deficiency in female mice, we cannot assess the relative contribution of DDX3Y to the innate immune response against Lm *in vivo*. Specifically, we cannot address a potential contribution of the DDX3 isoforms to the increased susceptibility of female C57BL/6 mice noted by others [50,54]. Bone-marrow derived macrophages from *Ddx3x*^*fl/fl CreERT2*^ mice, however, allowed us to compare expression levels of innate immune genes induced upon Lm infection of cells representing both sexes. This approach again revealed an incomplete rescue by DDX3Y. It correlated well with the strongly altered gene expression of infected macrophages *in vitro*. Since macrophages are ubiquitous primary targets of Lm in infected tissues they are major players in shaping the local cytokine milieu and, concomitantly, the activation status of surrounding cells. Consistent with this notion macrophages lacking one or both DDX3 isoforms showed a significant impairment in limiting the growth of intracellular Lm. Activation by IFNγ increased their antibacterial activity, but not to the same level observed in control cells. Thus, reduced cell-autonomous immunity as a consequence of less activating stimuli in the extracellular environment is likely to cause an impairment of mice to reduce the bacterial burden by innate mechanisms. The data in S1 Fig show that the broad effect observed upon DDX3X deficiency particularly in female macrophages did not translate into a similarly widespread impact on serum cytokine levels in infected mice. On the one hand this may result from the fact that male mice had to be used for infection experiments, hence from the weaker impact of DDX3X deficiency in cells expressing DDX3Y. Alternatively, macrophages may not be responsible for building up high levels of serum cytokines.

In summary, loss of DDX3X impacts on the innate immune system both through its role in hematopoiesis and its regulation of the innate response to Listeria infections. It alters lymphoid organ cellularity and the cytokine cocktail needed for cell recruitment and functional activation of innate immune cells.

## Materials and methods

### Ethics statement

Animal experiments were carried out at the University of Veterinary Medicine Vienna and have been approved by the institutional ethics and animal welfare committee and the national authority (Austrian Federal Ministry of education, Science and Research) according to §§26ff of Animal Experiments Act (Tierversuchsgesetz TVG 2012, BGBl. I Nr 114/2012) under the permission license numbers BMWF 68.205/0032-WF/II/3b/2014 and BMWFW-68.205/0212-WF/V/3b/2016. Animal husbandry and experimentation was performed under the Austrian national law and the ethics committees of the University of Veterinary Medicine Vienna and according to the guidelines of FELASA which match those of ARRIVE.

### Cells

Mouse embryonic fibroblast (MEFs) deficient in either TBK1/IKKε (kindly provided by S. Akira, Osaka University, Osaka, Japan, or in DDX3X (derived from *Ddx3x*^*fl/y CreERT2*^ or *Ddx3x*^*fl/fl CreERT2*^ embryos and immortalized by the 3T3 method), as well as HEK293 cells (American Type Culture Collection CRL-3216) were cultured in DMEM (Sigma, distributed by Sigma-Aldrich Handels GmbH, Vienna, Austria) supplemented with 10% FBS (Sigma, distributed by Sigma-Aldrich Handels GmbH, Vienna, Austria) and with penicillin and streptomycin (Sigma, distributed by Sigma-Aldrich Handels GmbH, Vienna, Austria). Deletion of DDX3X was carried out by Tamoxifen treatment (4-OHT; Sigma, distributed by Sigma-Aldrich Handels GmbH, Vienna, Austria) for 48 hours at a final concentration of 500nM.

### Plasmids

The plasmids pIE-NHA-hDDX3X, pIE-NHA-hMAVS, pCS2-N-Myc-mTBK1 and pEF-HA-mIRF3 were described previously [4]. The expression vector pIE-NHA-DDX3Y was generated based on the RefSeq ID: NM_012008.2 using the *EcoRI* and *BspeI* restriction sites. FLAG-IRF7-M15 [40] was obtained from Isabelle Marié (New York University, NY, USA). The NFκB-reporter plasmid was kindly provided by Ann J. Richmond (Vanderbilt University School of Medicine, Nashville, Tennessee, USA) [55].

### Antibodies

Anti-GAPDH (Clone ABS16; Millipore GesmbH, Vienna, Austria) was used in 1:3000 dilution; anti-DDX3X (Clone A300-474A; Bethyl Laboratories Inc., distributed by Sanova Pharma, Vienna, Austria) was used in 1:1000 dilution; anti-HA.11 Epitope Tag (Clone MMS-101P-200; Cambridge Bioscience, Cambridge, UK) was used in 1:1000 dilution; anti-Myc (Clone 9B11; CST, distributed by New England Biolabs, Frankfurt am Main, Germany) was used in 1:1000 dilution; anti-Flag (anti-ESC; Clone A190-101A; Bethyl Laboratories Inc., distributed by Sanova Pharma, Vienna, Austria) was used in 1:1000 dilution.

### Flow cytometry

Viability stains were carried out according to manufacturer's protocol (Fixable Viability Dyes; formerly ebioscience, now Thermo Fisher Scientific, distributed by Fisher Scientific GmbH, Vienna, Austria). Cells were pre-incubated with TruStain fcX (formerly ebioscience, now Thermo Fisher Scientific, distributed by Fisher Scientific Austria GmbH, Vienna, Austria) and stained with appropriate surface antibodies in PBS 2% FCS. For intracellular cytokine stainings, cells were fixed in 2% PFA and permeabilized using a saponin-containing buffer (BD Perm/Wash; Becton Dickinson Austria GmbH, Schwechat, Austria). Anti-CD11b (Clone M1/70), anti-CD3e (Clone 145-2C11), anti-CD8a (Clone 53–6.7), anti-B220 (Clone RA3-6B2), anti-Ly6G (Clone 18A), anti-CD11c (Clone HL3), anti-CD49b (Clone Dx5) anti-CD44 (Clone IM7), anti-Gr1 (Clone RB6-8C5), anti-Ter119 (Clone TER119) and anti-CD122 (Clone 5H4) antibodies were purchased from BD Bioscience (Becton Dickinson Austria GmbH, Schwechat, Austria) and used according to manufacturer's instructions. Anti-CD4 (Clone GK1.5), anti-NK1.1 (Clone PK136), anti-NKp46 (Clone 29A1.4), anti-IFNγ (Clone XMG1.2), anti-F4/80 (Clone BM8), anti-Ly6C (Clone HK1.4), anti-BP1 (Clone 6C3), anti-CD43 (Clone ebioR2/60), anti-IgM (Clone eB121-15F9), anti-CD24 (Clone M1/69), anti-cKit (Clone 2B8), anti-Sca1 (Clone D7) and anti-CD127 (Clone A7R34) were purchased from Thermo Fisher Scientific (formerly ebioscience, now distributed by Fisher Scientific Austria GmbH, Vienna, Austria) and used according to manufacturer's instructions. PBS-57 loaded and PE-conjugated CD1d tetramers were obtained from NIH Tetramer Core facility (NIH Tetramer Core Facility at Emory University, Atlanta, US) and used according to manufacturer's instructions. Fluorochrome labelled Annexin V and 7-AAD were purchased from Thermo Fisher Scientific (formerly ebioscience, now distributed by Fisher Scientific Austria GmbH, Vienna, Austria) and used according to manufacturer's instructions. Data was acquired on BD FACSAria or BD LSRFortessa and analyzed using FlowJo Software.

### Cell culture, infection, transfection and stimulation

MEFs were transiently transfected in 6-well plates (3,5x 10^5^ cells/well) using Turbofect (Thermo Fisher Scientific, distributed by Fisher Scientific Austria GmbH, Vienna, Austria) according to manufacturer’s instructions. DNA amount across the samples was equalized by transfecting an empty vector. Bone-marrow derived macrophages were obtained from *Ddx3x*^*fl/fl CreERT2*^ and *Ddx3x*^*fl/y CreERT2*^ mice via flushing the tibia and femur. Macrophages were differentiated in DMEM (10% FBS, pen/strep) containing recombinant M-CSF (a kind gift from L. Ziegler-Heitbrock, Helmholtz Center, Munich, Ger). On day 5, medium was changed to starvation medium (DMEM, 2% FBS, pen/strep, M-CSF) and half of the cells was treated with 4-OHT (Sigma, distributed by Sigma-Aldrich Handels GmbH, Vienna, Austria) for 48 hours at a final concentration of 500nM to induce deletion of *Ddx3x*. After 48 hours medium was replaced (DMEM, 10% FBS, pen/strep, M-CSF). On day 8–9 cells were seeded for experiments. BMDMs were stimulated with 30 μg/ml poly (I:C) (formerly Amersham Biosciences, now GE Healthcare Services Europe, distributed by Fischer Scientific Austria GmbH, Vienna, Austria) or 10 μg/ml poly (dA:dT) (Sigma, distributed by Sigma-Aldrich Handels GmbH, Vienna, Austria) using Hyperfect (Qiagen, Vienna, Austria) and Polyfect (Qiagen, Vienna, Austria) respectively according to the manufacturer’s instructions. LPS (*Escherichia coli* 055:B5; Sigma, distributed by Sigma-Aldrich Handels GmbH, Vienna, Austria) was added dropwise to a final concentration of 100ng/ml. Where indicated, cells were treated with IFNβ (PBL, distributed by Enzo Life Sciences, Lörrach, Germany) at a final concentration of 250U/ml or IFNγ (formerly ebioscience, now Thermo Fisher Scientific, distributed by Fisher Scientific Austria GmbH, Vienna, Austria) at a final concentration of 5ng/ml. *L*. *monocytogenes* strain LO28 was grown for 16 hours in brain heart infusion medium (Becton Dickinson Austria GmbH, Schwechat, Austria). BMDMs (seeded the day before in antibiotic free DMEM supplemented with 10% FBS) were infected at a multiplicity of infection (MOI) of 10 for 1 h at 37°C. After this, cells were washed with PBS and complete DMEM containing 50 μg/ml gentamicin (MP Biomedicals, Santa Ana, US) was added to kill extracellular bacteria. After another 1 h, BMDMs were washed with PBS again, and medium was replaced with DMEM containing 10 μg/ml gentamicin and left for another 2 hours. After stimulation, BMDMs were washed two times with PBS and used for RNA isolation.

### siRNA-mediated gene knockdown

MEFs were transfected with either 50pmol DDY3Y siRNA or with 50pmol non-targeting siRNA (Dharmacon, distributed by THP Medical Products GmbH, Vienna) using Lipofectamine RNAiMAX (Thermo Fisher Scientific, distributed by Fisher Scientific Austria GmbH, Vienna, Austria) under antibiotic-free conditions. After 48 hours, medium was exchanged and the cells transfected for 4 hours with 10μg/ml poly (dA:dT) (Sigma, distributed by Sigma-Aldrich Handels GmbH, Vienna, Austria) using Polyfect (Qiagen, Vienna, Austria) according to the manufacturer’s instructions. Subsequently, cells were washed once with PBS and used for RNA isolation.

### Mice

Floxed Ddx3x (Ddx3x^fl/fl^ or DDX3X^fl/y^) mice were generated via homologous recombination in ES cells. Specifically, loxP sites flanking exon 2 were introduced by gene targeting. *Ddx3x*^*fl/fl CreERT2*^, *Ddx3x*^*fl/y CreERT2*^ and *Ddx3x*^*fl/y Vav-iCre*^ mice on a C57BL/6 genetic background were housed under specific pathogen-free conditions according to FELASA guidelines. NK cell depletion of *Ddx3x*^*fl/y*^, *Ddx3x*^*fl/y Vav-iCre*^ and C57BL/6 mice was carried out by injecting the anti-Nk1.1 antibody (150 μg/mouse) 3 days prior to infection. Depletion efficiency was confirmed by flow cytometry of blood and splenic leukocytes. For infection experiments an overnight culture of *Listeria monocytogenes* was recultured in BHI medium to late logarithmic phase, pelleted and diluted in PBS. The concentration of *L*. *monocytogenes* was quantified by optical density measurements at 600 nm. The infectious dose was controlled by plating serial dilutions on BHI agar plates and counting the colonies. For infection, bacteria were diluted in PBS and 200 μl were injected into the peritoneum of 8- to 12-week-old mice. For the infection with VSV, mice were infected i.v. with 100 μl of 1x106 plaque forming unit (pfu) of VSV. The progress of the disease was monitored every 2–4 h during the “day phase” (7 a.m. to 7 p.m.) or both during the “day” and the “night phase” depending on the condition of the animals. In survival or terminal stage experiments, humane endpoint by cervical dislocation was conducted if death of the animals was expected within next few hours.

### Infection of MEFs with VSV and plaque forming unit (pfu) assay

MEFs were infected with VSV at an MOI of 0,1 under FCS-free conditions. After 1 hour of infection, the medium was exchanged to remove unbound virus. After the indicated periods of time, the supernatants were collected. On the next day, L-929 cells (American Type Culture Collection CCL-1) cells were infected by a dilution series prepared from the collected supernatants in DMEM. After 1 hour of infection, the medium was replaced with DMEM containing 0,5% low melting point (LMP) agarose (Thermo Fisher Scientific, distributed by Fisher Scientific Austria GmbH, Vienna, Austria). After 24 hours, crystal violet solution (Sigma, distributed by Sigma-Aldrich Handels GmbH, Vienna, Austria) was added onto the agarose to stain the cells. Subsequently, plaques were counted and the viral titer was determined.

### RNA-Seq

Bone-marrow-derived macrophages were infected with *L*. *monocytogenes* strain LO28 as described above. Additionally, BMDMs were stimulated with the combination of heat-killed Listeria (MOI: 50) and IFNβ (250U/mL) for 4hours. RNA was extracted from bone-marrow-derived macrophages using the NucleoSpin RNA II kit (Macherey-Nagel, distributed by VWR International GmbH, Vienna, Austria). RNA samples were poly(A) selected, libraries were prepared using TruSeq RNA Sample Preparation Kit (Illumina Inc., San Diego, US) as per manufacturers instruction, and sequenced (50 bp single-end read) on an Illumina HiSeq 2000. Sequencing data have been deposited to NCBI Gene Expression Omnibus and are accessible through GEO series accession number GSE86591.50-bp single-end Illumina mRNA sequencing reads were aligned to the mm10 reference genome using STAR (v 2.5.0a), and gene-level read counts were obtained using htseq-count (HTSeq v 0.6.1p1) [56]. Heat map of log 2-fold gene expression changes between Listeria-treated and untreated samples were generated for genes differentially expressed upon treatment in WT samples of either sex (padj <0.01, abs(logFC)>1; DeSeq2 v1.18.1). Functional enrichment analysis of the differentially expressed genes using DAVID v6.8- top 10 enriched GO terms ranked by p-value for the category GOTERM_BP_3 are shown [57].

### Colony forming unit assay

The assay was performed a recently described [39]. Briefly, 5x10^4^ BMDMs were seeded into 96-well plates in DMEM (10% FBS, no antibiotics) and stimulated with IFNγ as indicated. On the next day, an overnight culture of *L*. *monocytogenes* strain LO28 was used to infect BMDMs at an MOI of 10. After uptake of the bacteria medium containing gentamicin was added to kill extracellular bacteria. BMDMs were washed two times with PBS followed by lysis in dH2O at the indicated time points. Serial dilutions of the lysates were plated onto brain heart infusion plates followed by incubation for one day at 37°C. Intracellular bacteria were quantified by counting the number of colonies. Data shown are representatives of at least 3 independent experiments.

### Western blot analysis

Proteins were isolated during RNA isolation with NucleoSpin RNA kit (Macherey-Nagel, distributed by VWR International GmbH, Vienna, Austria). In brief, after adjusting RNA binding conditions with 70% ethanol, lysate were centrifuged. At this step nucleic acids are bound to the silica membrane of the column, whereas proteins flow through. After collecting the flow-through containing the proteins, equal amounts of Protein Precipitator (Macherey-Nagel, distributed by VWR International GmbH, Vienna, Austria) was added and samples were left at room temperature for 10 minutes. After this, samples were centrifuged at 11.000g for 15 minutes and supernatants were discarded. Precipitated proteins were washed with 50% ethanol and centrifuged again. Supernatants were discarded, pellets were dryed and 30 μl of 1x Laemmli sample buffer (62,6 mM Tris pH:6,8, 10% Glycerol, 2% SDS, 7,1% β-mercaptoethanol, bromophenol blue) was added to them. The samples were then subjected to western blot analysis as described above. After primary antibody binding the blots were probed with fluorescence-labelled secondary antibodies (formerly Invitrogen, now Thermo Fisher Scientific, distributed by Fisher Scientific Austria GmbH, Vienna, Austria) at a dilution of 1:15000 and detected by the Odyssey infrared imaging system (LI-COR, Lincoln, NE).

### Determination of serum cytokines

For cytokine analysis blood was taken by cardiac puncture and serum was isolated via centrifugation at 10.000g for 1 minute. Cytokine concentrations were determined using the FlowCytomix system (formerly ebioscience, now Thermo Fisher Scientific, distributed by Fisher Scientific Austria GmbH, Vienna, Austria). In some experiments, IFNγ concentrations were measured by ELISA (formerly ebioscience, now Thermo Fisher Scientific, distributed by Fisher Scientific Austria GmbH, Vienna, Austria).

### RNA isolation, cDNA synthesis and real-time quantitative PCR

Total RNA was extracted from mouse embryonic fibroblasts and bone-marrow-derived macrophages using the NucleoSpin RNA II kit (Macherey-Nagel, distributed by VWR International GmbH, Vienna, Austria). The cDNAs was prepared using Oligo (dT_18_) Primer and the RevertAid Reverse Transcriptase (Thermo Fisher Scientific, distributed by Fisher Scientific Austria GmbH, Vienna, Austria). Real-time qPCR experiments were run on an Eppendorf Mastercycler to amplify the *Gapdh* (housekeeping gene), using SybrGreen (Promega, Mannheim, Germany). Real-time qPCR assays targeting mRNA of *Gapdh*, *Ifnb*, *Il-1b*, *Il-6*, *Il-12p40*, *Nos2*, *Tnfα*, *Irf1*, *Mx1*, *Isg15*, *Ifit3* and *Oas2* and pre-mRNA of *Il-1b*, *Il-6*, *Nos2*, *Tnfα*, *Ccl5* and *Cxcl10* were performed with the following forward (f) and reverse (r) primers: mRNA: *Gapdh*-f: 5’-CATGGCCTTCCGTGTTCCTA-3’; *Gapdh*-r: 5’-GCGGCACGTCAGATCCA-3’; *Ifnb*-f: 5’-TCAGAATGAGTGGTGGTTGC-3’; *Ifnb*-r: 5’-GACCTTTCAAATGCAGTAGATTCA-3’; *Il-1b*-f: 5’-AGATGAAGGGCTGCTTCCAAA-3’; *Il-1b*-r: 5’-AATGGGAACGTCACACACCA-3’;, *Il-6*-f: 5’-CTGCAAGAGACTTCCATCCAG-3’, *Il-6*-r: 5’-AGTGGTATAGACAGGTCTGTTGG-3’; *Il-12p40*-f: 5’-TGGTTTGCCATCGTTTTGCTG-3’; *Il-12p40*-r: 5’-ACAGGTGAGGTTCACTGTTTCT-3’; *Nos2*-f: 5’- GAGCAACTACTGCTGGTGGT-3’; *Nos2*-r: 5’- CGATGTCATGAGCAAAGGCG-3’, *Tnfα*-f: 5’- CAAAATTCGAGTGACAAGCCTG-3’; *Tnfα*-r: 5’- GAGATCCATGCCGTTGCC-3’; *Irf1-f*:5'-CCG AAG ACC TTA TGA AGC TCT TTG-3' *Irf1-r*: 5'-GCA AGT ATC CCT TGC CAT CG-3'; *Mx1-f*: 5'-GAC TAC CAC TGA GAT GAC CCA GC-3', *Mx1-r*: 5'-ATT TCC TCC CCA AAT GTT TTC A-3'; *Isg15-f*: 5'-ATG GCC TGG GAC CTA AAG-3'; *Isg15-r*: 5'-TTA GGC ACA CTG GTC CCC-3'; *Ifit3-f*: 5'-CCT CGC AGC CCT GGA GTG TT-3'; *Ifit3-r*: 5'-TGC GTT GCC TCC CAA ACC CC-3'; *Oas2-f*: 5'-AAACCTCACACCCAACGAAAA-3'; *Oas2-r*: 5'- CCACCCTTAGCCACTTCCT-3'. pre-mRNA: *Il-1b*-f: 5’-AAGATGAAGGTGAGACTCTGAG-3’; *Il-1b*-r: 5’-CTTGGTGTGTGGCTGTGGTA-3’; *Il-6*-f: 5’-AATGGAGTTGTTAGGCATGGG-3’; *Il-6*-r: 5’-TGTAAATCTTTTACCTAAAGGAGGA-3’; *Nos2*-f: 5’-TCCTTTAAAGAGTAAGTCTGGCTT-3’; *Nos2*-r: 5’-CAGGACTCAGCAGTGACCT-3’; *Tnfα*-f: 5’-AGGGATGAGGTGAGTGTCTG-3’; *Tnfα*-r: 5’-ACGTGTGAACACACTTGTTCGT-3’; *Ccl5*-f: 5’-GCCTCACCATGTAAGTCGAG-3’; *Ccl5*-r: 5’-CACAGAAAAGTTCCTCAGAGGA-3’; *Cxcl10*-f: 5’-TGGGACTCAAGGTAAGGGAC-3’; *Cxcl10*-r: 5’-CTTTCTTCCCTTCTTCGTTCCT-3’.

### Dual luciferase assay

Luciferase activity was measured with the Dual Luciferase Reporter Assay System (Promega, Mannheim, Germany). In short, the cell lysates were mixed with the substrate for the Firefly luciferase, then luciferase activity was determined with the luminometer. After this, the stop solution ending the first enzymatic reaction and containing the substrate for the Renilla luciferase was added and the samples were measured again. Quantification was performed normalizing the Firefly luciferase activity to the Renilla luciferase activity. Relative induction levels were derived comparing the samples to an empty control vector.

### Statistical analysis

Bacterial loads in spleens and livers were compared using unpaired *t*-test. The lines represent mean with the standard error of the mean (SEM). Bacterial loads in *in vitro* CFU assays were compared with the unpaired *t*-test and bars on the graph represent the mean values with standard deviation (SD). Significances of survival curves after either Lm or VSV infections were calculated using the Mantel-cox-test. Serum cytokine levels were compared using the unpaired *t*-test where the lines represent means with the standard error of the mean. The mRNA expression data are represented with the mean values with standard deviation (SD). The differences in mRNA expression data were compared using the paired *t*-test. All statistical analyses were performed using the GraphPad Prism (GraphPad) software. Asterisks denote statistical significance as follows: ns, P > 0.05; *P ≤ 0.05; **P ≤ 0.01; ***P ≤ 0.001.

## Supporting information

S1 Fig Serum cytokines in mice infected with *Listeria monocytogenes*. *Ddx3x*^fl/y^ mice (n = 3 [day1] and n = 13 [day3]) and *Ddx3x*^fl/y *Vav-iCre*^ mice (n = 3 [day1] and n = 14 [day3]) were infected intraperitoneally with 1x10^5^ CFU *L*. *monocytogenes* (strain EGD) for the indicated periods of time. Mice were sacrificed and serum was collected. Cytokine levels indicated on the x-axes were analyzed by flow cytometry-based bead array. Statistical significance was calculated using the unpaired, two-tailed Student's *t*-test. *P<0.05, **P<0.01, ***P<0.005.
(TIF)

S1 Table Genes differentially expressed (padj <0.01, logFC>1; DeSeq2 v1.18.1) in Listeria-infected (l) and control macrophages (c) are displayed in the following order: Female Wt, Male wt, female DDX3X-/- and male DDX3X-/y. Male and female macrophages were derived from *Ddx3x*^fl/y Cre*ERT2*^ male and *Ddx3x*^fl/fl;Cre*ERT2*^ female mice, respectively. Cells were treated with 4-OHT to delete DDX3X.
(XLS)

## References

[1] Goubau D, Deddouche S, Reis E Sousa C. Cytosolic sensing of viruses. Immunity. 2013;38: 855–869. 10.1016/j.immuni.2013.05.007 23706667 10.1016/j.immuni.2013.05.007 PMC7111113

[2] Paludan SR, Bowie AG. Immune sensing of DNA. Immunity. 2013;38: 870–880. 10.1016/j.immuni.2013.05.004 23706668 10.1016/j.immuni.2013.05.004 PMC3683625

[3] Stavrou S, Aguilera AN, Blouch K, Ross SR. DDX41 Recognizes RNA/DNA Retroviral Reverse Transcripts and Is Critical for In Vivo Control of Murine Leukemia Virus Infection. MBio. American Society for Microbiology; 2018;9: e00923–18. 10.1128/mBio.00923-18 29871919 10.1128/mBio.00923-18 PMC5989071

[4] Soulat D, Burckstummer T, Westermayer S, Goncalves A, Bauch A, Stefanovic A, et al The DEAD-box helicase DDX3X is a critical component of the TANK-binding kinase 1-dependent innate immune response. EMBO J. 2008; 27: 2135–2146. 10.1038/emboj.2008.126 18583960 10.1038/emboj.2008.126 PMC2453059

[5] Schröder M, Baran M, Bowie AG. Viral targeting of DEAD box protein 3 reveals its role in TBK1/IKKepsilon-mediated IRF activation. EMBO J. 2008; 27: 2147–2157. 10.1038/emboj.2008.143 18636090 10.1038/emboj.2008.143 PMC2516890

[6] Fairman-Williams ME, Guenther U-P, Jankowsky E. SF1 and SF2 helicases: family matters. Curr Opin Struct Biol. 2010; 20: 313–324. 10.1016/j.sbi.2010.03.011 20456941 10.1016/j.sbi.2010.03.011 PMC2916977

[7] Linder P, Jankowsky E. From unwinding to clamping—the DEAD box RNA helicase family. Nat Rev Mol Cell Biol. 2011;12: 505–516. 10.1038/nrm3154 21779027 10.1038/nrm3154

[8] Fuller-Pace FV. DExD/H box RNA helicases: multifunctional proteins with important roles in transcriptional regulation. Nucleic Acids Res. 2006; 34: 4206–4215. 10.1093/nar/gkl460 16935882 10.1093/nar/gkl460 PMC1616952

[9] Bol GM, Xie M, Raman V. DDX3, a potential target for cancer treatment. Mol Cancer. BioMed Central; 2015;14: 188 10.1186/s12943-015-0461-7 10.1186/s12943-015-0461-7 PMC4636063 26541825

[10] Chen C-Y, Chan C-H, Chen C-M, Tsai Y-S, Tsai T-Y, Wu Lee Y-H, et al Targeted inactivation of murine Ddx3x: essential roles of Ddx3x in placentation and embryogenesis Human molecular genetics. Oxford University Press; 2016;: ddw143 10.1093/hmg/ddw143 10.1093/hmg/ddw143 27179789

[11] Li Q, Zhang P, Zhang C, Wang Y, Wan R, Yang Y. DDX3X regulates cell survival and cell cycle during mouse early embryonic development. J Biomed Res. 2014 28(4): 282–291. 10.7555/JBR.27.20130047 25050112 10.7555/JBR.27.20130047 PMC4102842

[12] Ditton HJ, Zimmer J, Kamp C, Rajpert-De Meyts E, Vogt PH. The AZFa gene DBY (DDX3Y) is widely transcribed but the protein is limited to the male germ cells by translation control. Human molecular genetics. 2004 ed. 2004;13: 2333–2341. 10.1093/hmg/ddh240 15294876 10.1093/hmg/ddh240

[13] Sekiguchi T, Iida H, Fukumura J, Nishimoto T. Human DDX3Y, the Y-encoded isoform of RNA helicase DDX3, rescues a hamster temperature-sensitive ET24 mutant cell line with a DDX3X mutation. Exp Cell Res. 2004 ed. 2004;300: 213–222. 10.1016/j.yexcr.2004.07.005 15383328 10.1016/j.yexcr.2004.07.005

[14] Fish EN. The X-files in immunity: sex-based differences predispose immune responses. Nature Rev Immunol. 2008;8: 737–744. 10.1038/nri2394 18728636 10.1038/nri2394 PMC7097214

[15] Casimir GJ, Lefèvre N, Corazza F, Duchateau J. Sex and inflammation in respiratory diseases: a clinical viewpoint. Biol Sex Differ. BioMed Central; 2013;4: 16 10.1186/2042-6410-4-16 24128344 10.1186/2042-6410-4-16 PMC3765878

[16] Klein SL, Flanagan KL. Sex differences in immune responses. Nature Rev Immunol. 2016;16: 626–638. 10.1038/nri.2016.90 27546235 10.1038/nri.2016.90

[17] Lai M-C, Sun HS, Wang S-W, Tarn W-Y. DDX3 functions in antiviral innate immunity through translational control of PACT. FEBS J. 2016;283: 88–101. 10.1111/febs.13553 26454002 10.1111/febs.13553 PMC7164078

[18] Li Q, Brass AL, Ng A, Hu Z, Xavier RJ, Liang TJ, et al A genome-wide genetic screen for host factors required for hepatitis C virus propagation. Proc Natl Acad Sci USA; 2009;106: 16410–16415. 10.1073/pnas.0907439106 19717417 10.1073/pnas.0907439106 PMC2752535

[19] Oshiumi H, Ikeda M, Matsumoto M, Watanabe A, Takeuchi O, Akira S, et al Hepatitis C virus core protein abrogates the DDX3 function that enhances IPS-1-mediated IFN-beta induction. Coers J, editor. PLoS ONE. Public Library of Science; 2010;5: e14258 10.1371/journal.pone.0014258 21170385 10.1371/journal.pone.0014258 PMC2999533

[20] Mahboobi SH, Javanpour AA, Mofrad MRK. The Interaction of RNA Helicase DDX3 with HIV-1 Rev-CRM1-RanGTP Complex during the HIV Replication Cycle. Marcello A, editor. PLoS ONE. Public Library of Science; 2015;10: e0112969 10.1371/journal.pone.0112969 25723178 10.1371/journal.pone.0112969 PMC4344243

[21] Rifo RS, Rubilar PS, Limousin T, de Breyne S, Décimo D, Ohlmann T. DEAD‐box protein DDX3 associates with eIF4F to promote translation of selected mRNAs. EMBO J. 2012; 31: 3745–3756. 10.1038/emboj.2012.220 22872150 10.1038/emboj.2012.220 PMC3442272

[22] Lai M-C, Wang S-W, Cheng L, Tarn W-Y, Tsai S-J, Sun HS. Human DDX3 Interacts with the HIV-1 Tat Protein to Facilitate Viral mRNA Translation. PLoS ONE. Public Library of Science; 2013;8: e68665 10.1371/journal.pone.0068665 23840900 10.1371/journal.pone.0068665 PMC3698215

[23] Valiente Echeverría F, Hermoso MA, Soto Rifo R. RNA helicase DDX3: at the crossroad of viral replication and antiviral immunity. Reviews in Medical Virology. 2015;25: 286–299. 10.1002/rmv.1845 26174373 10.1002/rmv.1845

[24] Fullam A, Schröder M. DExD/H-box RNA helicases as mediators of anti-viral innate immunity and essential host factors for viral replication. Biochimica et Biophysica Acta (BBA)—Gene Regulatory Mechanisms. 2013;1829: 854–865. 10.1016/j.bbagrm.2013.03.012 23567047 10.1016/j.bbagrm.2013.03.012 PMC7157912

[25] Oshiumi H, Sakai K, Matsumoto M, Seya T. DEAD/H BOX 3 (DDX3) helicase binds the RIG-I adaptor IPS-1 to up-regulate IFN-β-inducing potential. Eur J Immunol. 2010;40: 940–948. 10.1002/eji.200940203 20127681 10.1002/eji.200940203

[26] Wu X, Dao Thi VL, Huang Y, Billerbeck E, Saha D, Hoffmann H-H, et al Intrinsic Immunity Shapes Viral Resistance of Stem Cells. Cell. 2018;172: 423–438.e25. 10.1016/j.cell.2017.11.018 29249360 10.1016/j.cell.2017.11.018 PMC5786493

[27] Decker T, Muller M, Stockinger S. The yin and yang of type I interferon activity in bacterial infection. Nature Rev Immunol. 2005;5: 675–687. 10.1038/nri1684 16110316 10.1038/nri1684

[28] Dussurget O, Pizarro-Cerda J, Cossart P. Molecular determinants of Listeria monocytogenes virulence. Annu Rev Microbiol. 2004 ed. 2004;58: 587–610. 10.1146/annurev.micro.57.030502.090934 15487949 10.1146/annurev.micro.57.030502.090934

[29] Pamer EG. Immune responses to Listeria monocytogenes. Nature Rev Immunol. 2004;4: 812–823. 10.1016/S0140-6736(06)68772-210.1038/nri1461 15459672 10.1038/nri1461

[30] Sauer J-D, Sotelo-Troha K, Moltke von J, Monroe KM, Rae CS, Brubaker SW, et al The N-ethyl-N-nitrosourea-induced Goldenticket mouse mutant reveals an essential function of Sting in the in vivo interferon response to Listeria monocytogenes and cyclic dinucleotides. Infection and Immunity. 2011;79: 688–694. 10.1128/IAI.00999-10 21098106 10.1128/IAI.00999-10 PMC3028833

[31] Hansen K, Prabakaran T, Laustsen A, Jørgensen SE, Rahbæk SH, Jensen SB, et al Listeria monocytogenes induces IFNβ expression through an IFI16-, cGAS- and STING-dependent pathway. EMBO J. 2014;33: 1654–1666. doi: 10.15252/embj.201488029 24970844 10.15252/embj.201488029 PMC4194099

[32] Abdullah Z, Schlee M, Roth S, Mraheil MA, Barchet W, Böttcher J, et al RIG-I detects infection with live Listeria by sensing secreted bacterial nucleic acids. EMBO J. 2012;31: 4153–4164. 10.1038/emboj.2012.274 23064150 10.1038/emboj.2012.274 PMC3492734

[33] Carrero JA, Calderon B, Unanue ER. Type I interferon sensitizes lymphocytes to apoptosis and reduces resistance to Listeria infection. J Exp Med. 2004;200: 535–540. 10.1084/jem.20040769 15302900 10.1084/jem.20040769 PMC2211931

[34] Auerbuch V, Brockstedt DG, Meyer-Morse N, O'Riordan M, Portnoy DA. Mice lacking the type I interferon receptor are resistant to Listeria monocytogenes. J Exp Med. 2004; 200: 527–533. 10.1084/jem.20040976 15302899 10.1084/jem.20040976 PMC2211930

[35] Stockinger S, Kastner R, Kernbauer E, Pilz A, Westermayer S, Reutterer B, et al Characterization of the interferon-producing cell in mice infected with Listeria monocytogenes. Philpott DJ, editor. PLoS pathogens. 2009 ed. 2009;5: e1000355 10.1371/journal.ppat.1000355 19325882 10.1371/journal.ppat.1000355 PMC2654726

[36] Huang S, Hendriks W, Althage A, Hemmi S, Bluethmann H, Kamijo R, et al Immune response in mice that lack the interferon-gamma receptor. Science (New York, NY. 1993;259: 1742–1745. 10.1126/science.8456301 8456301 10.1126/science.8456301

[37] Harty JT, Bevan MJ. Specific immunity to Listeria monocytogenes in the absence of IFN gamma. Immunity. 1995;3: 109–117. 10.1016/1074-7613(95)90163-9 7621071 10.1016/1074-7613(95)90163-9

[38] Humann J, Lenz LL. Activation of Naive NK Cells in Response to Listeria monocytogenes Requires IL-18 and Contact with Infected Dendritic Cells. J Immunol. American Association of Immunologists; 2010;184: 5172–5178. 10.4049/jimmunol.0903759 20351186 10.4049/jimmunol.0903759 PMC2920760

[39] Kernbauer E, Maier V, Stoiber D, Strobl B, Schneckenleithner C, Sexl V, et al Conditional Stat1 Ablation Reveals the Importance of Interferon Signaling for Immunity to Listeria monocytogenes Infection. PLoS pathogens. Public Library of Science; 2012;8: e1002763 10.1371/journal.ppat.1002763 22719255 10.1371/journal.ppat.1002763 PMC3375314

[40] Caillaud A, Hovanessian AG, Levy DE, Marie IJ. Regulatory serine residues mediate phosphorylation-dependent and phosphorylation-independent activation of interferon regulatory factor 7. J Biol Chem. 2005 ed. 2005;280: 17671–17677. 10.1074/jbc.M411389200 15743772 10.1074/jbc.M411389200 PMC1224706

[41] Barchet W, Cella M, Odermatt B, Asselin-Paturel C, Colonna M, Kalinke U. Virus-induced interferon alpha production by a dendritic cell subset in the absence of feedback signaling in vivo. J Exp Med. 2002;195: 507–516. 10.1084/jem.20011666 11854363 10.1084/jem.20011666 PMC2193622

[42] Frenz T, Graalmann L, Detje CN, Döring M, Grabski E, Scheu S, et al Independent of plasmacytoid dendritic cell (pDC) infection, pDC triggered by virus-infected cells mount enhanced type I IFN responses of different composition as opposed to pDC stimulated with free virus. J Immunol. American Association of Immunologists; 2014;193: 2496–2503. 10.4049/jimmunol.1400215 25070849 10.4049/jimmunol.1400215

[43] Tripp CS, Wolf SF, Unanue ER. Interleukin 12 and tumor necrosis factor alpha are costimulators of interferon gamma production by natural killer cells in severe combined immunodeficiency mice with listeriosis, and interleukin 10 is a physiologic antagonist. Proc Natl Acad Sci USA. 1993rd ed. 1993;90: 3725–3729. 8097322 10.1073/pnas.90.8.3725 PMC46374

[44] Berg RE, Cordes CJ, Forman J. Contribution of CD8+ T cells to innate immunity: IFN‐γ secretion induced by IL‐12 and IL‐18. Eur J Immunol.; 2002;32: 2807–2816. 10.1002/1521-4141(2002010)32:10<2807::AID-IMMU2807>3.0.CO;2-0 12355433 10.1002/1521-4141(2002010)32:10<2807::AID-IMMU2807>3.0.CO;2-0

[45] Ranson T, Bregenholt S, Lehuen A, Gaillot O, Leite-de-Moraes MC, Herbelin A, et al Invariant Vα14+ NKT Cells Participate in the Early Response to Enteric Listeria monocytogenes Infection. J Immunol. American Association of Immunologists; 2005;175: 1137–1144. 10.4049/jimmunol.175.2.1137 16002715 10.4049/jimmunol.175.2.1137

[46] Tripp CS, Gately MK, Hakimi J, Ling P, Unanue ER. Neutralization of IL-12 decreases resistance to Listeria in SCID and C.B-17 mice. Reversal by IFN-gamma. J Immunol. American Association of Immunologists; 1994;152: 1883–1887. 7907107

[47] Dai WJ, Bartens W, Kohler G, Hufnagel M, Kopf M, Brombacher F. Impaired macrophage listericidal and cytokine activities are responsible for the rapid death of Listeria monocytogenes-infected IFN-gamma receptor-deficient mice. J Immunol. 1997 ed. 1997;158: 5297–5304. 9164949

[48] Andersson A, Dai WJ, Di Santo JP, Brombacher F. Early IFN-gamma production and innate immunity during Listeria monocytogenes infection in the absence of NK cells. J Immunol. 1998;161: 5600–5606. 9820538

[49] Clark SE, Filak HC, Guthrie BS, Schmidt RL, Jamieson A, Merkel P, et al Bacterial Manipulation of NK Cell Regulatory Activity Increases Susceptibility to Listeria monocytogenes Infection. PLoS pathogens. Public Library of Science; 2016;12: e1005708 10.1371/journal.ppat.1005708 10.1371/journal.ppat.1005708 PMC4905663 27295349

[50] Pasche B, Kalaydjiev S, Franz TJ, Kremmer E, Gailus-Durner V, Fuchs H, et al Sex-dependent susceptibility to Listeria monocytogenes infection is mediated by differential interleukin-10 production. Infection and Immunity.; 2005;73: 5952–5960. 10.1128/IAI.73.9.5952-5960.2005 16113316 10.1128/IAI.73.9.5952-5960.2005 PMC1231091

[51] Shi C, Hohl TM, Leiner I, Equinda MJ, Fan X, Pamer EG. Ly6G+ neutrophils are dispensable for defense against systemic Listeria monocytogenes infection. J Immunol. 2011;187: 5293–5298. 10.4049/jimmunol.1101721 21976773 10.4049/jimmunol.1101721 PMC3208088

[52] Wang T, Birsoy K, Hughes NW, Krupczak KM, Post Y, Wei JJ, et al Identification and characterization of essential genes in the human genome. Science; 2015;350: 1096–1101. 10.1126/science.aac7041 26472758 10.1126/science.aac7041 PMC4662922

[53] Knott GJ, Bond CS, Fox AH. The DBHS proteins SFPQ, NONO and PSPC1: a multipurpose molecular scaffold. Nucleic Acids Res. Oxford University Press; 2016;44: 3989–4004. 10.1093/nar/gkw271 27084935 10.1093/nar/gkw271 PMC4872119

[54] Yeretssian G, Doiron K, Shao W, Leavitt BR, Hayden MR, Nicholson DW, et al Gender differences in expression of the human caspase-12 long variant determines susceptibility to Listeria monocytogenes infection. Proc Natl Acad Sci USA.; 2009;106: 9016–9020. 10.1073/pnas.0813362106 19447924 10.1073/pnas.0813362106 PMC2690057

[55] Yang J, Richmond AJ. Chapter 17 Monitoring NF‐κB Mediated Chemokine Transcription in Tumorigenesis. Chemokines, Part A. Elsevier; 2009 pp. 347–355. 10.1016/S0076-6879(09)05217-3 PMC3140415 19446734

[56] Love MI, Huber W, Anders S. Moderated estimation of fold change and dispersion for RNA-seq data with DESeq2. Genome biology. BioMed Central; 2014;15: 550 10.1186/s13059-014-0550-8 10.1186/s13059-014-0550-8 PMC4302049 25516281

[57] Huang DW, Sherman BT, Lempicki RA. Systematic and integrative analysis of large gene lists using DAVID bioinformatics resources. Nature Protocols. Nature Publishing Group; 2009;4: 44–57. 10.1038/nprot.2008.211 19131956 10.1038/nprot.2008.211

