# Supplementary figures and images for: The RNA helicase DDX3X is an essential mediator of innate antimicrobial immunity
Source: PLoS Pathog. 2018 Nov 26;14(11):e1007397. doi: 10.1371/journal.ppat.1007397 (PMC6283616; doi:10.1371/journal.ppat.1007397)

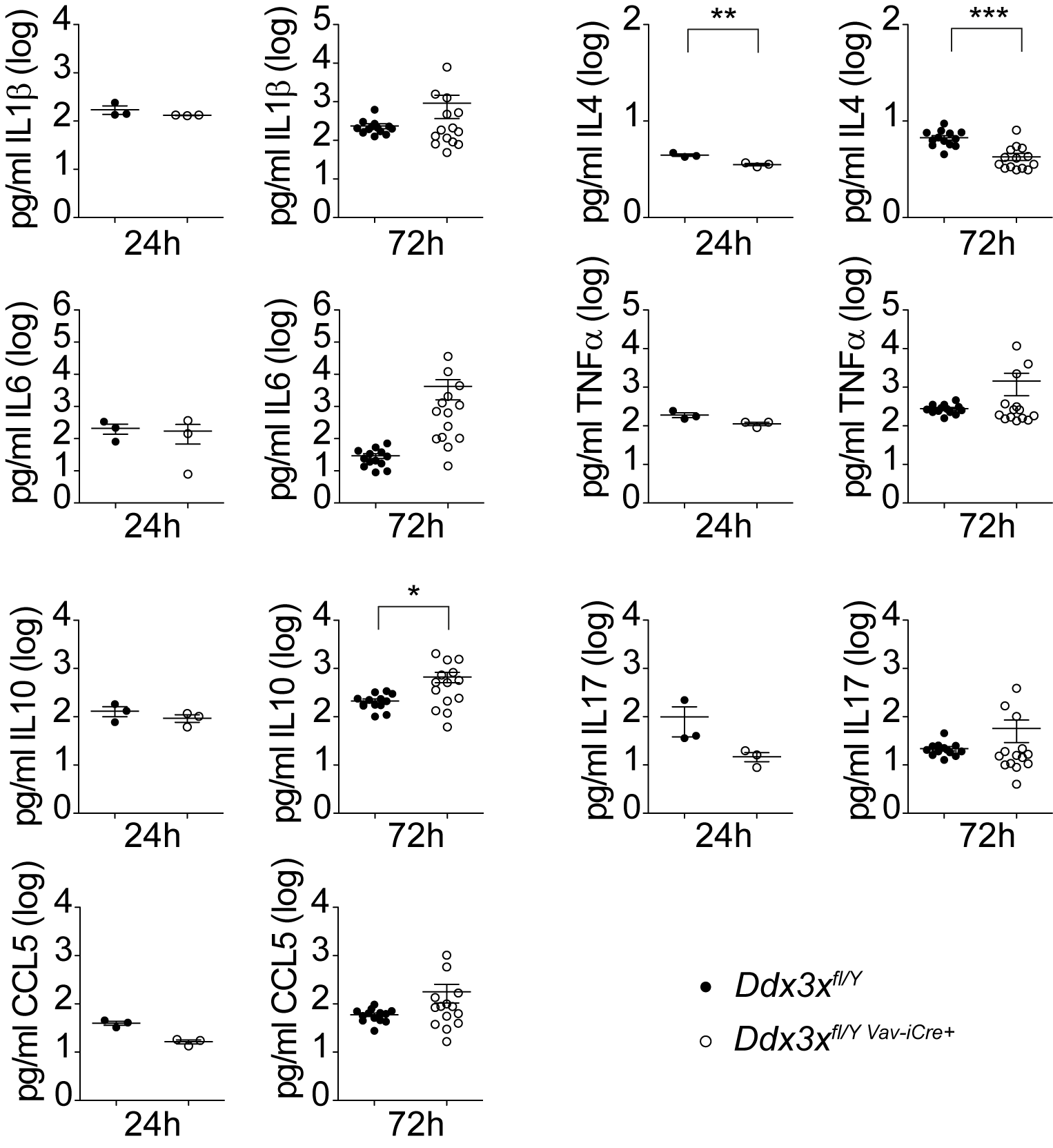

Supplement: S1 Fig — Ddx3xfl/y mice (n = 3 [day1] and n = 13 [day3]) and Ddx3xfl/y Vav-iCre mice (n = 3 [day1] and n = 14 [day3]) were infected intraperitoneally with 1x10^5^ CFU L. monocytogenes (strain EGD) for the indicated periods of time. Mice were sacrificed and serum was collected. Cytokine levels indicated on the x-axes were analyzed by flow cytometry-based bead array. Statistical significance was calculated using the unpaired, two-tailed Student's t-test. *P<0.05, **P<0.01, ***P<0.005. (TIF) [file ppat.1007397.s001.tif]
